# Link prediction and feature relevance in knowledge networks: A machine learning approach

**DOI:** 10.1371/journal.pone.0290018

**Published:** 2023-11-30

**Authors:** Antonio Zinilli, Giovanni Cerulli

**Affiliations:** IRCRES-Research Institute on Sustainable Economic Growth, CNR-National Research Council, Rome, Italy; Pontificia Universidad Catolica de Chile, CHILE

## Abstract

We propose a supervised machine learning approach to predict partnership formation between universities. We focus on successful joint R&D projects funded by the Horizon 2020 programme in three research domains: Social Sciences and Humanities, Physical and Engineering Sciences, and Life Sciences. We perform two related analyses: link formation prediction, and feature importance detection. In predicting link formation, we consider two settings: one including all features, both exogenous (pertaining to the node) and endogenous (pertaining to the network); and one including only exogenous features (thus removing the network attributes of the nodes). Using out-of-sample cross-validated accuracy, we obtain 91% prediction accuracy when both types of attributes are used, and around 67% when using only the exogenous ones. This proves that partnership predictive power is on average 24% larger for universities already incumbent in the programme than for newcomers (for which network attributes are clearly unknown). As for feature importance, by computing super-learner average partial effects and elasticities, we find that the endogenous attributes are the most relevant in affecting the probability to generate a link, and observe a largely negative elasticity of the link probability to feature changes, fairly uniform across attributes and domains.

## Introduction

Link prediction is a key element to describe the evolution of complex networks. Depending on the context of application, complex networks represent architectures characterized by highly nonlinear interactions among entities such as neural, physical, and social actors. As the importance of networks expands, understanding networks’ evolution, change, and endogenous dynamics is a key point. Therefore, developing efficient algorithms to study networks’ evolution scenarios has become a challenging task, as it involves a large set of interlinked actors and factors.

Link prediction for prospective collaborations among entities belonging to a scientific environment has attracted an increasing research interest [1, 2].

Based on past networks’ observation, this paper aims at contributing to the literature on knowledge networks by carrying out an accurate prediction of prospective links that *could* potentially take place among diverse academic entities operating in a specific knowledge context. More specifically, we consider project-based R&D collaborations taking place within the Horizon 2020 research funding programme and falling within the three European Research Council domains (or ERC classification): Social Sciences and Humanities (SSH), Physical and Engineering Sciences (PE), and Life Sciences (LS).

These three networks pursuit specific and different modes of knowledge creation, as diverse is their logic of activating collaborative research and the decision on how and with whom knowledge has to be exchanged. The link prediction problem in a competitive environment, as the Horizon 2020 programme, is therefore an entry point for examining the partnerships in the three above-mentioned areas. We are aware that collaborations can have powerful effects on scientific performance, and for this reason we aim at understanding the architecture and characteristics of the three scientific fields, and which factors (endogenous and exogenous to the network) may explain the link creation in one domain but not in the others.

Another motivation for conducting this research is in relation to the Horizon 2020 programme. We know that the funding for this programme is set aside to encourage collaboration through joint projects. In this regard, it is critical that the network is inclusive and accessible to new participants. This study aims at understanding to what extent being or not being already embedded in a certain network (i.e., being either an *incumbent*, or a *new-comer*) helps to predict future collaborations by assessing link prediction accuracy. We estimate link prediction accuracy considering all the features together at first (that is, those that are both exogenous and endogenous to the network), to then omitting the endogenous ones (that is, those features that belong to the observed network).

Indeed, for newcomers (e.g. actors that were not part of the observed network), we only know exogenous characteristics and not, for example, their centrality within the network (which is an endogenous-to-the-network feature). By disentangling the contribution of endogenous and exogenous components, we are able to measure to what extent link prediction is accurate for the incumbents as well as for the newcomers. As a result, link prediction among Horizon 2020 actors is critical, particularly in the light of the Europe 2030 initiative. Related to this, there is an ongoing debate in the literature about how such collaborations can increase R&D productivity [3, 4].

By means of recent developments in machine learning predictive algorithms, in this paper we attempt to estimate the *probability* that a university *A* collaborates with a university *B* by considering their idiosyncratic attributes, as well as the past centrality and the sharing of common neighbors. For this purpose, we attempt to achieve a high prediction accuracy at a reasonable computational cost.

This paper has a twofold objective: on the one hand, as a methodological contribution, it supports the necessity to employ/compare different supervised machine learning algorithms for link prediction; on the other hand, as a policy objective, it aims at providing decision-makers with a new perspective on how to assess the dynamics of the collaborations among the EU academic organizations, thereby promoting effective integration of the actors belonging to the European Research Area (ERA).

We look at the link prediction problem with the double lens of the network theory [5, 6] as our theoretical background, and machine learning [7–9] as our data-driven approach.

Links’ creation takes place through different mechanisms. Some of these rely on the similarity concept that is operationally based on nodes’ similar characteristics [10–12]; another frequent mechanism described in the literature is the so-called “preferential attachment” [13, 14]: two nodes have a higher probability to form a connection if they own common neighbors or similar features [5, 10]. Some mechanisms are based on past topological structures, such as cyclical forms, and hierarchical structures [15, 16], whereas others are based on node’s centrality as a measure affecting both the static and dynamic processes of a network formation and development [17]. For a more comprehensive review on network link formation and prediction, one can refer to Kerrache et al [18] and Lü and Zhou [19].

Most of the previous literature focuses on nodes and topological network characteristics. In this paper, we take on a wider perspective. Indeed, as university collaboration networks in joint research projects are composed of heterogeneous information, our assumption is that their formation is driven by topological (information stemming from the graph adjacency matrix), as well as non-topological node features (node attributes improving prediction accuracy).

The paper is organized as follows. Section 2 provides a brief state-of-the-art of link prediction in a knowledge network context. Section 3 describes the database and methodology and presents our formulation of the link prediction. Section 4 sets out the results and their discussion. Section 5 closes the paper.

## A brief look at the extant literature

Most of the theoretical and applied studies in Network Science (NS) have focused on the underlying patterns of the process-building of connections among nodes [20–24]. In the last few years, a new wave of interest and research in the social network analysis has taken place by focusing on link prediction. Link prediction is in fact of the utmost relevance in several knowledge network sub-fields, including co-authorship networks [1, 2, 25], and future scientific impact of scholars [26, 27].

In this paper, we look at a specific knowledge network, namely the network of European universities that received funding in the three aforementioned ERC domains via the Horizon 2020 programme from 2014 to 2016. Focusing on three distinct domains allows us to identify differences with respect to the driving factors of link creation in a project funding scheme. By taking on an organizational perspective, we assume that academic participation in the Horizon 2020 programme is mainly driven by organizational factors, as well as the regional environment the universities lie on. Collaboration among academic organizations is an important lever of knowledge diffusion and acquisition, as each university is a network’s node linked to other nodes via joint projects.

In its focus, this study follows the steam of literature investigating structures and dynamics of knowledge networks in competitive project funding. In recent years, increased competition in the scientific ecosystem has led universities to act more and more strategically [28, 29]. As a consequence, there has been increased emphasis on promoting the autonomy of universities seeking funding streams different from the government core funds, such as project funding on competitive scaling [29, 30]. The access to several funding resources, and a greater prestige and reputation of the organization, lead to cumulative advantages sustained by preferential attachment mechanisms and the creation of closed groups [31]. Link prediction for knowledge networks is thus relevant, as it allows for the forecast of prospective collaborations among scientists and their organizations.

These collaborations make it possible to facilitate financing strategies on the part of policymakers, providing support for further improving future relationships among scientists and academic organizations. Unlike predicting the co-authorship network and the related impact of scholar activities, predicting joint project collaborations at the university level is relatively easier. Collaborations in joint projects, both at the scientist and organizational level, are in fact lasting longer time, thus leading to generate rarer changes during the years than it happens with co-authorship or citation networks [32]. Research on predicting joint project collaborations is still in its initial phase, and providing methods and techniques for predicting collaborations seems a promising area of research.

Many university features play an important role in the collaboration behavior. The size of a university is certainly relevant, as larger universities tend to attract more requests for collaborations [33, 34]. With regard to a specific italian project funding, Zinilli [24] pointed out that Betweenness centrality and geographical proximity are important drivers of collaboration formation. Newman [35] shows that scientists have a higher probability to collaborate if they have common neighbors. Abbasi et al [17] indicated the importance of centrality measures to explain preferential attachment in a scientific collaborative publication network. They have stressed the relevance of Betweenness centrality as predictor of the preferential attachment for a new node. Likewise, similarity seems to play an important role in link prediction, as nodes with similar features are likelier to get connected in the nearby future [11]. As result, global similarity-based indexes such as the Katz Index [36] are often used for predicting links in large networks [48]. Depending on the application, the importance of a vertex can have different meanings and hence several network centrality measures have been used, for instance degree, Closeness and Betweenness centrality. Analyzing citation networks by machine learning techniques, Shibata et al. [37] concluded that the Jaccard coefficient, the Betweenness centrality, and the cosine similarity are powerful factors affecting link prediction. This result is further bolstered by the recent study conducted by Resce, Zinilli, and Cerulli [38], which focuses on predicting linkages in coauthorships. In this research, we use the Jaccard, Katz Index (or centrality), Closeness and Betweenness centrality coefficients as endogenous (i.e., network-related) factors to explain network formation in the light of the previously cited literature. The Jaccard coefficient represents the similarity of the neighborhoods of two vertices. The idea behind neighborhood similarity as predictor of new link formation is that the presence of many common adjacent nodes between two nodes indicates a higher chance of new connection’s formation between those two nodes. The Jaccard coefficient between node *i* and *j*, is given by:

$$\begin{array}{c} J_{ij}=\frac{\left|N(i)\cap N(j)\right|}{\left|N(i)\cup N(j)\right|} \end{array}$$ (1)

The Katz index measures the closeness of two nodes in this network as the weighted sum of all pathways linking the two nodes, where the path weights collapse exponentially with path length [48].

$$\begin{array}{c} K_{i,j}=\sum_{l=1}^{\infty }\alpha^{l}paths_{l}\left(i,j\right) \end{array}$$ (2)

where *paths*_*l*_(*i*, *j*) denotes the number of paths of length *l* connecting (*i*) and (*j*) in a specific network.

Closeness centrality refers to the average shortest distance between any given two nodes. Formally, we define Closeness centrality, *C*_*ij*_, between two nodes (*i*) and (*j*) in a network with (*N*) nodes as follows:

$$\begin{array}{c} C_{ij}=\frac{N}{d_{i,j}} \end{array}$$ (3)

Note that (*d*_*i*,*j*_) is the shortest distance between the nodes (*i*) and (*j*).

The Betweenness centrality, *B*_*ij*_, is defined as the number of shortest paths passing through (*i*, *j*) among those linking all node pairs (*u*, *v*) of the network. Betweenness stresses those links that facilitate information exchange among network members. More specifically:

$$\begin{array}{c} B_{ij}=\sum_{u,v\in V}\frac{B_{u,v}^{ij}}{B_{u,v}} \end{array}$$ (4)

where *B*_*u*,*v*_ is the number of shortest paths connecting (*u*, *v*), and $B_{u,v}^{ij}$ is the number of such paths passing through (*i*, *j*).

As different studies have shown [e.g. 24, 31, 39], the collaboration in research projects could be explained by different factors, either endogenous (related to the network structure), and exogenous to the extant network (related to the node attributes). It is thus important to consider the endogenous characteristics of a graph (among others, the Jaccard coefficient, Katz, Closeness and the Betweenness centrality) along with the exogenous node attributes [18].

In what follows, we address three related questions: (i) to what extent can collaborations in joint projects be accurately predicted using machine learning algorithms, by jointly and separately considering endogenous and exogenous node characteristics? (ii) what is the role played by node topological features vis-à-vis link prediction? (iii) what features, either endogenous or exogenous to the network, have a larger impact in predicting new links, and in what direction do they act?

## Materials and methods

### Problem formulation

The key point of this paper is to predict the formation of network connections. For this purpose, it is important to combine factors that measure different aspects of the nodes into a single data structure. The issue can be articulated as follows: given a node pair *i* and *j*, where *i* and *j* may be (incumbents) or may not be (newcomers) part of the current network, we aim at predicting the probability of this pair to generate a relationship in the next future.

**Fig 1:**
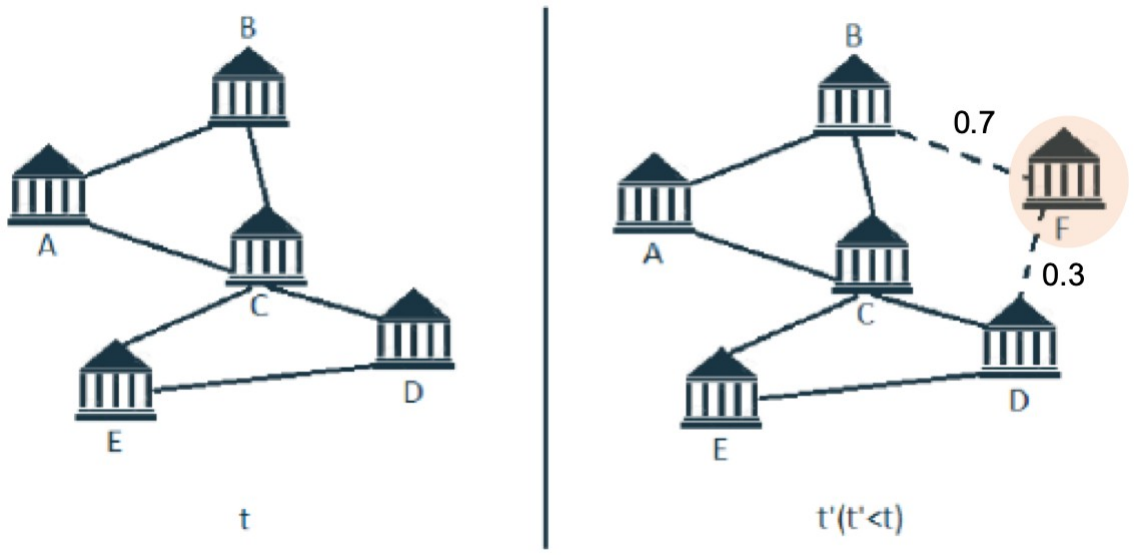
Representation of two graphs: Observed links at time *t*, and missing link prediction at time *t*′.

Suppose *G*(*N*, *L*) to be an undirected graph, with *N* a set of nodes (e.g. the universities funded under Horizon 2020 between 2014 and 2016 in the three ERC domains), and *L* representing the link between nodes. Loop and multiple links among nodes are not considered in this analysis. Our aim is to predict the link probability of a given node with respect to all the other nodes of the graph. Given a snapshot of the network at time *t*, we seek to predict what links are likely to be created (or, possibly, kept) in a future time *t*′. Fig 1 shows the observed links at time *t*, and the link prediction at time *t*′, considering a new node *F* that did not belong to the network at time *t*.

By means of popular machine learning algorithms, for each node in the network, we determine all the possible link probabilities with the other nodes. In the simplified example of the figure, node *F* has zero link probabilities with all the other nodes, except for node *B* and *D*. The link with node *B* has a 70% likelihood to occur, against a 30% of the link with node *D*. It is thus likelier that university *F* will collaborate with university *B* in a new round of the research funding programme herein analyzed. Of course, it is relevant to evaluate to what extent these probabilities are accurately estimated. This is the aim of the first part of our study (first question), where we set out the out-of-sample performance accuracy of such predictions using several machine learning predictive algorithms. The accuracy is clearly conditional on the features employed. As already said, in fact, the features can be endogenous to the nodes (e.g., Jaccard coefficient and Betweenness centrality), or exogenous to the nodes (e.g., the gross domestic product of the region a node belongs to).

The distinction between endogenous and exogenous features is relevant as it entails a prediction trade-off we aim at exploring in this paper (second question): indeed, if we use both endogenous and exogenous features, we are unable to predict link probabilities for the so-called *newcomers* in the programme, as these entities have no (past) information on network endogenous measures, as they have never been part of the extant network. In this case, we are obliged to rely only on those entities that are already part of the network, as for them we know both endogenous and exogenous features. This is a limitation, but it lends larger predictive power, as long as the endogenous features are highly correlated with link prediction (as a large empirical evidence supports). In contrast, relying only on exogenous features allow us for estimating link probabilities also for the newcomers, a task that may be of great interest for understanding future new configurations of the network. However, as in this case we are excluding from the analysis features possibly highly conducive to predict new links, the statistical accuracy of the estimated probabilities could be poorer, undermining the forecasting quality of the network evolution. Providing a measure of the accuracy reduction produced by excluding the endogenous features is one of the contribution of this study.

Finally, this paper provides a more in-depth understanding of the role played by each single feature in steering the probability of setting-up a link (third question). We carry out this analysis by aggregating over all the learners employed (“super-learning”) the *average partial effect* (APE) of each single feature. For a single feature *x*_*j*_, the APE is the derivative of the probability to lie a link with respect to the feature *x*_*j*_ itself, with all the other features kept fixed at their sample mean. As machine learning algorithms are highly nonparametric, this derivative is a highly nonlinear function of the level of the feature; exploring APE’s shape sheds light on the relative importance of each feature in driving link probability. Interestingly, beside derivatives, we also explore the pattern of the *elasticities* to catch the percentage change activated by a one percentage change in every considered feature.

### Data preprocessing and step-wise implementation

This paper presents a set of machine learning algorithms for predicting network links. Here, we discuss the different steps we have undertaken for the data preprocessing. Originally, we had a bipartite network (university by project), and the variables at university level. Then, we transformed the network into a one-mode format. The one-mode projection means a network containing all universities’ pairs. According to the ERC panels, we assigned each project to one the following three research domains: Social Sciences and Humanities (SSH), Physical Sciences and Engineering (PE) and Life Sciences (LS). Each project is assigned to a domain based on its topic identifier, keywords, and abstract content. Each project has a topic identifier that allows it to be assigned to an ERC domain. When the topic identifier for the project was ambiguous (no direct association), the project was assigned to a domain based on the content of its abstract. A small number of projects (just over 2% of the total) is excluded from the study because explicitly described as multidisciplinary, thus not having a clear attribution to a research domain. Because of their primarily individual nature, we also exclude Marie Curie and European Research Council projects. To prepare the dataset for running the machine learning algorithms, we separate the target variable and the features, with the features transformed into normally standardized variables. We only have one training dataset, and we measure learners’ goodness-of-fit via a 10-fold cross-validation out-of-sample accuracy. As accuracy measure, we employ the complement of the error rate, that is, the out-of-sample proportion of correct matches found between the actual and the predicted labels.

$$\begin{array}{c} \text{Accuracy}=1-\sum_{i=1}^{N}I\left[y_{i}\neq \hat{L}\left(X_{i}\right)\right] \end{array}$$ (5)

where: *N* is the number of universities’ pairs; *y*_*i*_ is the link binary variable for pair *i* (taking value 1 if a link exists, and 0 otherwise); $\hat{L}\left(\cdot \right)$ is a generic learner (a classifier, in our case); *X*_*i*_ is a vector of *p* features (either exogenous or endogenous); and *I*[⋅] is an indicator variable taking value 1 if the statement into the squared parenthesis is true, and 0 otherwise. Eq (5) can be estimated both in-sample (training accuracy), or out-of-sample (testing accuracy). As usual in machine learning, testing accuracy will be retained as our reference goodness-of-fit, being training accuracy plagued by overfitting).

Also, to take into account the large number of zeros within our link target variable, we carried out random under-sampling.

For carrying out our analysis along the line mentioned above, we proceed according to the following road-map:

1. We start using the EUPRO dataset (a dataset providing information on R&D projects, participants and resulting networks of the EU FPs) to build the R&D project network from 2014 (when the Horizon 2020 programme has started) to 2016. In our network, the node is the university, and the link indicates a collaboration on a joint project within the Horizon 2020 programme.
2. We classify projects according to the ERC panels, by assigning each project to a specific research domain: Social Sciences and Humanities, Physical Sciences and Engineering, and Life Sciences. The assignment is based on project keywords and on the content of the project’s abstract. When a project is multidisciplinary, with no clear designation of its domain, we exclude it from the analysis. In this way we are able to capture the specificity of each ERC domain. Given their larger specificity, we do not consider ERC grants and Marie Curie projects. To circumscribe our analysis to more homogeneous partnerships, we focus on the three ERC domains separately.
3. We use the RISIS-ETER (database on European Higher Education Institutions) database to extract university level variables and the geographical distance for each couple of universities, and combine EUPRO and RISIS-ETER information using the same node ID.
4. We use a proxy for university size based on the number of students belonging to a specific ERC domain, and compute the Jaccard coefficient, the Katz score and Closeness and Betweenness centrality for every node in every year.
5. We apply several supervised machine learning methods for predicting universities’ link probabilities. As our link target outcome is a binary factor variable (taking value 1 if there is a link, and 0 otherwise), we run several binary classification experiments.
6. We compare the performance of the proposed learning algorithms by jointly considering—for every learner—the average out-of-sample (or “test”) accuracy, and the standard deviation obtained by a 10-fold cross-validation resampling procedure.
7. We further compute the accuracy achieved by comparing two specifications of our predictive model, one embedding both exogenous and endogenous features, and one considering only exogenous features. We thus calculate the accuracy gap.
8. For each learner, we then estimate the average partial effects (APE) function, i.e. the derivative of the conditional probability function with respect to one feature, with all the others held fixed at their sample mean.
9. We aggregate all the derivatives obtained in the previous step by averaging over them, thus obtaining a super-learning derivative estimate that we plot, compare, and comment for every feature.
10. As a derivative measures, by definition, infinitesimal changes, we also calculate *elasticities* to assess the percentage change of link probability induced by a given percentage change in the considered feature.

The features employed for link prediction are:

- EXOGENOUS FEATURES
  - *Regional gross domestic product* (PPS per inhabitant), measured at regional level from 2014 to 2016. Universities located in regions with a higher gross domestic product (GDP) are likely to hold the necessary capacities and resources to acquire public funding for collaborative projects [40].
  - *Core funding*, indicating the overall government funding available for a university. It consists mainly of basic government allocation. It can be considered both as a magnitude of financial inputs in knowledge production, and as a “boost” to push universities to increasingly raise funding (e.g., by participating to competitive projects) [41]. It is thus a lever to help generating additional (external) funding.
  - *Citation score*, measured by the “mean normalized citation score”, is the average number of citations of a university’s publications, normalized by field differences and publication year. This variable is a proxy of university reputation. Citations have been widely used in the scientific literature to capture knowledge outputs [see, for example, 42].
  - *Number of students by ERC domain*, considered as a proxy of university size re-scaled within the three ERC domains.
  - *Inverse of the distance*, considered as the Euclidean distance in terms of kilometers between two uniersities. We used the inverse of geographical distance as a measure of proximity between universities. The inverse of geographical distance can be interpreted as a measure of closeness or connectedness between universities, where larger values indicate stronger proximity.
- ENDOGENOUS FEATURES
  - *Jaccard coefficient*, defined as the proportion of common neighbours in the total number of neighbours. This index is maximum when neighbours are common to both nodes. In co-authorship networks, the Jaccard coefficient catches the idea that nodes having common neighbors are likelier to connect with each other in the next future. In joint project networks this occurrence is however not always true [43].
  - *Katz centrality* is determined by summing up the contributions of all its neighbors and their neighbors, with each contribution weighted by a factor of decay. Universities with high Katz scores are those that not only have direct collaborations with other universities but also have connections to other universities through their collaborative partners.
  - *Closeness centrality*, like Betweenness centrality, is a distance-based measure, reflecting the importance of nodes according to their connection distances in the network. It reflects how easily or quickly a node can reach other nodes in terms of geodesic distance (the shortest path between two nodes). Universities with high Closeness centrality scores are considered more central as they have shorter average distances to other universities in the network.
  - *Betweenness centrality*, referring to the frequency that a university acts as a connection between a pair of other universities. It is computed for all universities every year. A university with higher Betweenness has a larger influence on the whole network and can determine the network’s ability to capture resources and information. Universities with high Betweenness can benefit more from shorter paths towards a larger set of nodes as they are strongly embedded within the network structure.

### Optimal prediction via machine learning

We define a learner *L*_*j*_ as a mapping from the set [*X*, *θ*_*j*_, λ_*j*_, *f*_*j*_(⋅)] to an outcome *y*, where *X* is the matrix of features, *θ*_*j*_ a vector of estimation parameters, λ_*j*_ a vector of tuning parameters, and *f*_*j*_(⋅) an algorithm taking as inputs *X*, *θ*_*j*_, and λ_*j*_. Generally, applied empirical studies use a singleton *f*_*j*_(⋅) for modeling and predicting targeted outcomes, typically one member of the Generalized Linear Models (GLM) family (linear, probit or multinomial regressions are classical examples). GLM are highly parametric and are not characterized by tuning parameters. Nonparametric models, such as local-kernel, nearest-neighbor, or decision trees are on the contrary characterized by one or more hyper-parameters λ_*j*_ which may be optimally chosen to minimize the so-called *test prediction error*, i.e. the out-of-sample predicting accuracy of the learner.

**Fig 2:**
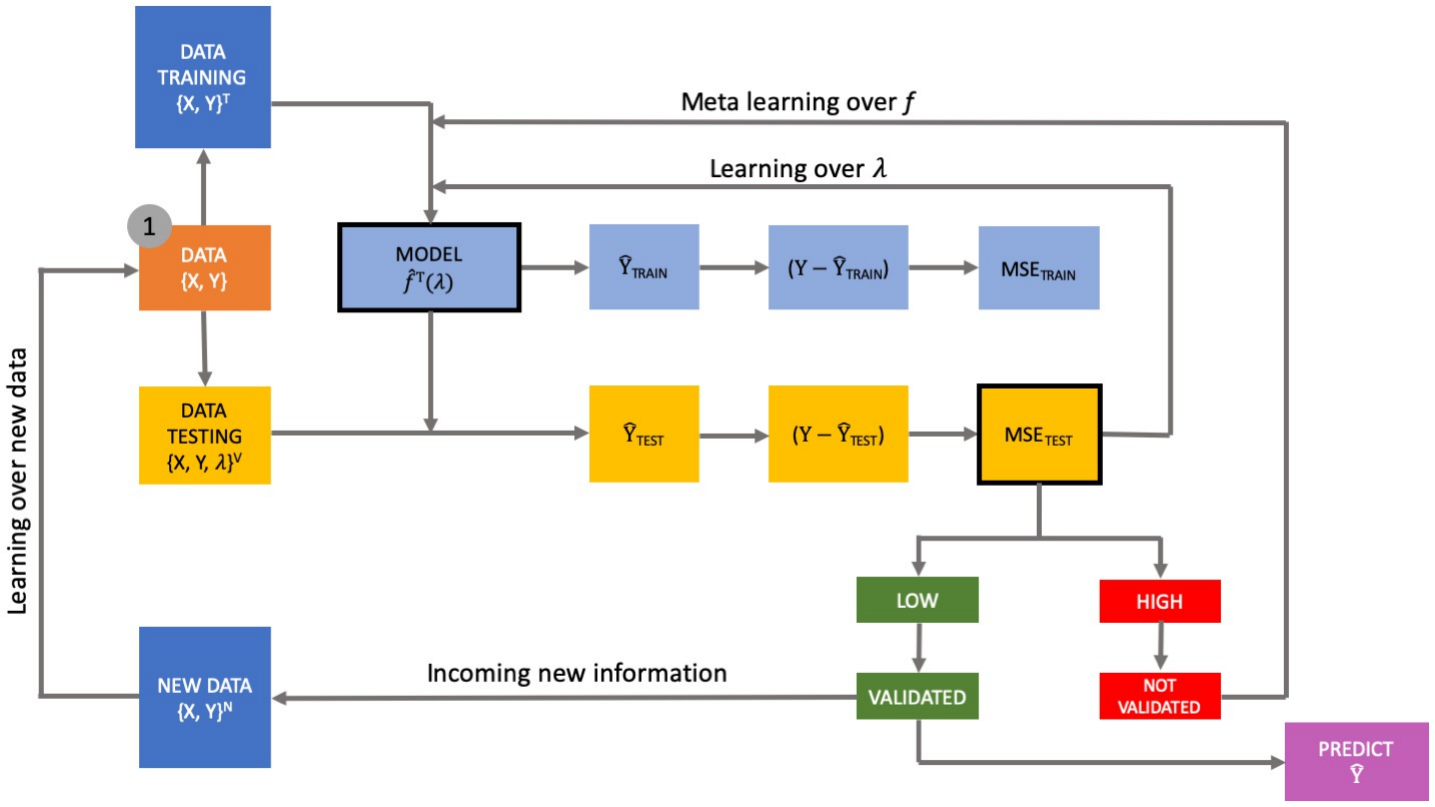
The meta-learning machine architecture.

Fig 2 presents the learning architecture herein proposed. This framework is made of three linked learning processes: (i) the learning over the tuning parameter λ, (ii) the learning over the algorithm *f*(⋅), and (iii) the learning over new additional information. The departure is in point 1, from where we set off assuming the availability of a dataset [*X*, *y*].

The first learning process aims at selecting the optimal tuning parameter(s) for a given algorithm *f*_*j*_(⋅). ML scholars typically do it using *K*-fold cross-validation, a resampling approach estimating the out-of-sample performance of a learner by leaving one group of observations out of the estimation, and then using prediction over these left-out observations to measure predictive accuracy. This procedure is iteratively repeated for each fold, eventually obtaining *K* test-accuracy (or, equivalently, test error) measures over which taking the average and the standard deviation.

At the optimal λ_*j*_, one can recover the largest possible prediction accuracy for the learner *f*_*j*_(⋅). Further prediction improvements can be achieved only by learning from other learners, namely, by exploring other *f*_*j*_(⋅), with *j* = 1, …, *M* (where *M* is the number of learners at hand).

It is important to observe that the so-called *training error*, i.e. the in-sample predictive performance of a learner, is a misleading measure of the actual model goodness-of-fit as plagued by the *overfitting* phenomenon: it may be the case that the training error decreases monotonically with the tuning parameter even if the out-of-sample performance of the learner is worsening. In Fig 2, it corresponds to the light blue sequence of boxes leading to the *MSE*_*TRAIN*_ which is in fact a dead-end node, as not informative for making correct decisions.

Conversely, the yellow sequence leads to the *MSE*_*TEST*_, which is informative to take correct decisions about the predicting quality of the current learner. At this node, the analyst can compare the current *MSE*_*TEST*_ with a benchmark one (possibly, pre-fixed), and conclude whether to predict using the current learner, or explore alternative learners in the hope of increasing predictive performance. If the level of the current prediction error is too high, the learning architecture would suggest to explore other learners.

In the ML literature, learning over learners is called *meta learning*, and entails an exploration of the out-of-sample performance of alternative algorithms *f*_*j*_(⋅) with the goal of identifying one behaving better than the those already explored [44]. For each new *f*_*j*_(⋅), the learning architecture finds an optimal tuning parameter and a new estimated accuracy (along with its standard deviation). The analyst can either explore the entire bundle of alternatives and finally pick-up the best one, or decide to select the first learner whose accuracy is larger than the benchmark. Either cases are automatically run by this architecture.

The third final learning process concerns the availability of new information, via additional data collection. This induces a reiteration of the initial process whose final outcome can lead to choose a different algorithm and tuning parameter(s), depending on the nature of the incoming information.

As final step, one may combine predictions of single optimal learners into one single super-prediction (*ensemble learning*). What is the advantage of this procedure? An aggregation of different learners, by reducing prediction variance, can lead to a smaller predictive uncertainty, thus improving overall prediction quality [45].

## Results

We run a series of popular machine learning algorithms to predict future link formation between universities. In particular, we compare the following learners: Tree, Random forest, Boosting, Regularized multinomial, Nearest neighbor, Neural network, Naïve Bayes, and Support vector machine.

We provide a brief description of these classification methods:

1. *Tree*. Decision tree algorithms are a type of supervised machine learning algorithm that partitions the input data based on a series of hierarchical decisions or rules. Each decision splits the data into two branches (recursive binary splitting algorithm) until reaching leaf nodes. Decision tree use the simple arithmetic mean to predict the target variable within each terminal node.
2. *Random forest*. Random forest is an ensemble learning method that combines multiple decision trees. Each tree is trained on a bootstrapped sample of the original data using a random subset of the features used as splitting variables at each node. The final prediction is obtained by aggregating the predictions of all individual trees, generally resulting in a more robust and accurate prediction.
3. *Boosting*. Boosting is another ensemble learning technique that combines multiple trees to create a strong predictive model. Boosting algorithms sequentially train models, with each subsequent model focusing on the instances that the previous models misclassified. Each boosted tree learns form the error and the final prediction is a weighted combination of the predictions from all the trees.
4. *Regularized multinomial*. Regularized multinomial algorithms are used for multiclass classification problems. These algorithms apply regularization techniques, such as L1 or L2 regularization, to prevent overfitting and improve generalization. They learn a set of coefficients for each class and use them to predict the target variable. Estimation is made using maximum likelihood.
5. *Nearest neighbor*. The nearest neighbor algorithm is a simple yet effective method for classification and regression. It works by finding the *k* nearest neighbors to a given data point and predicting the target variable based on the values of those neighbors (generally, a weighted mean). The choice of neighbors and the method used to calculate proximity (e.g., Euclidean distance) may vary.
6. *Neural network*. Neural networks are powerful models inspired by the structure and functioning of biological brains. They consist of interconnected nodes or artificial neurons organized in layers. Each neuron applies an activation function to its input, and the network learns by adjusting the weights between neurons through a process called *backpropagation*. Neural networks can be used for a wide range of tasks, including classification, regression, and pattern recognition. In our application, we consider only 2-layer networks.
7. *Naïve Bayes*. Naïve Bayes algorithms are based on Bayes’ theorem and assume that the features are conditionally independent in each class. They are commonly used for classification tasks, calculating the probability of a data point belonging to each class based on the feature values. Naïve Bayes algorithms are computationally fast and work well even with high-dimensional data.
8. *Support vector machine*: Support vector machines (SVMs) are supervised learning models used for classification as well as regression tasks. SVMs aim to find an optimal hyperplane that separates the data into different classes or predicts a continuous target variable. They maximize the margin between the closest data points of different classes, making them robust to outliers. SVMs can also use kernel functions to handle nonlinear relationships between features.

For each method, the optimal tuning of hyper-parameters have been found via a 10-fold cross-validation. Different ML methods have different tuning parameters, with methods like Tree having one singleton parameter—i.e. the number of final layers (otherwise known as *tree depth*), and methods like Boosting having three tuning parameters (namely: *tree depth*, *learning rate*, and *number of sequential trees, or iterations*). Learner-by-learner, Table 1 reports the list of the relevant tuning parameters.

**Table 1:** List of the relevant tuning parameters by learner.

LEARNER	λ_1_	λ_2_	λ_3_
Decision Tree	Leaves	–	–
Support Vector Machine	*C*	Γ	–
Regularized Multinomial	Penalization	–	–
Random Forest	Splitting features	Bootstraps	Tree–depth
Neural Network	Hidden layers	Neurons	–
Nearest Neighbor	Neighbors	–	–
Naïve Bayes	–	–	–
Boosting	Learning rate	Iterations	Tree–depth

In this work we do not consider deep-learning predicting algorithms. In the social sciences and business analytics data context, deep learning models have been proved to have predictive accuracy generally comparable to other ML methods [46]. This depends on the fact that deep-learning models improve prediction by exploiting some kind of *ordering* in the data. Convolutional neural networks, for example, exploit hierarchical spatial ordering in predicting images, while recurrent neural networks exploit sequential ordering to predict sequences (for example, time series). These two types of ordering are not present in our data, as in many other typical social sciences datasets [47]. For this reason, we preferred to stick to more comparable ML models, although we also estimated a neural network although in its fully-connected 2-layer structure course, there exists also a literature on link prediction based on deep-learning techniques. For example, Pan, Shi, and Dokmanic [48] propose an algorithm for network link prediction based on a random-walk pooling mechanism (called WalkPool) able to accurately learn network topological heuristics. In another paper, Pan et al. [49] proposed a novel deep-learning adversarial algorithm with two variants—adversarially regularized graph autoencoder (ARGA) and adversarially regularized variational graph autoencoder (ARVGA). The experimental results demonstrate the good performance of these algorithms on link prediction and other tasks. See also Wang et al. [50].

**Fig 3:**
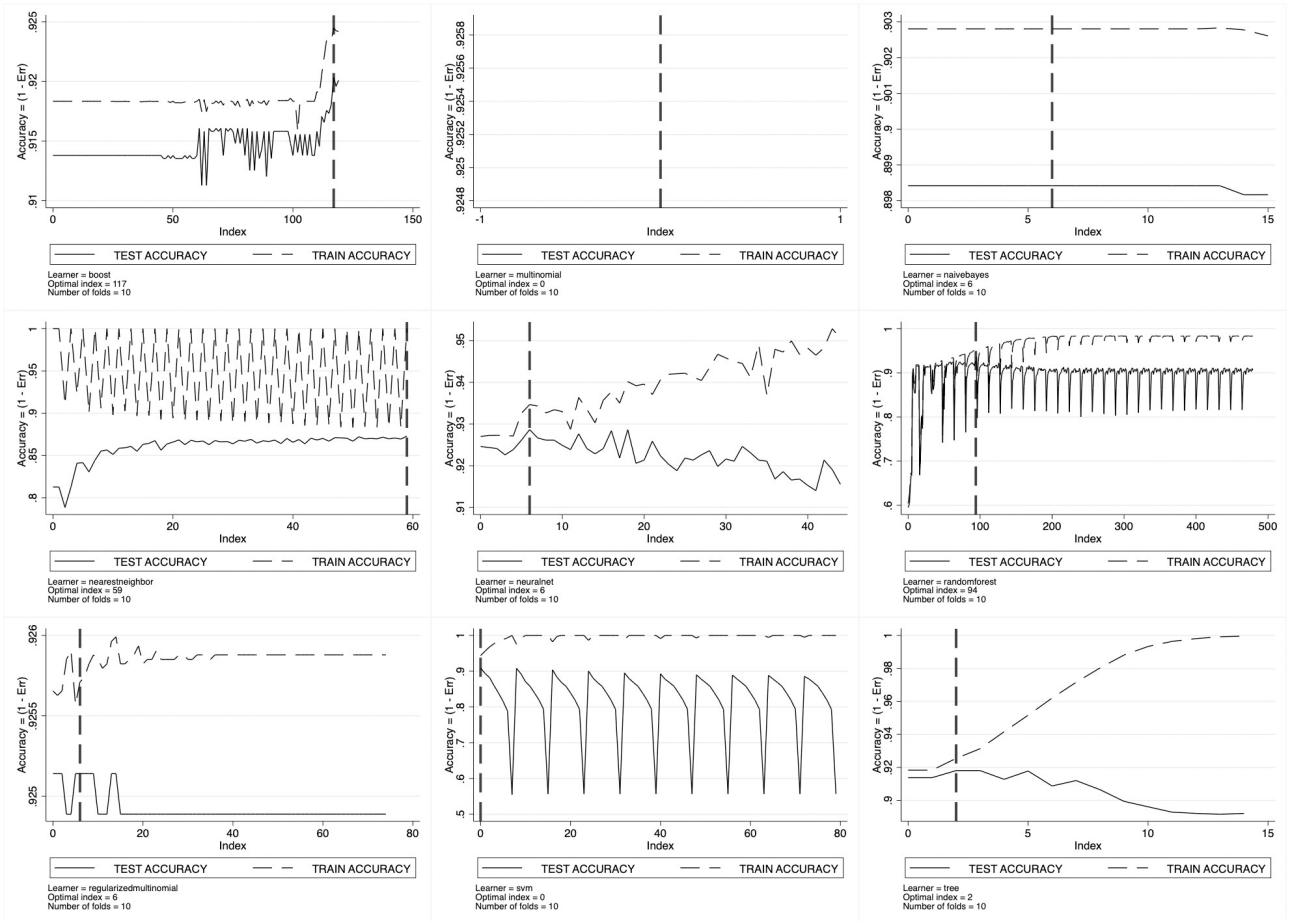
Learners’ optimal tuning using 10-fold cross-validation for SSH.

**Fig 4:**
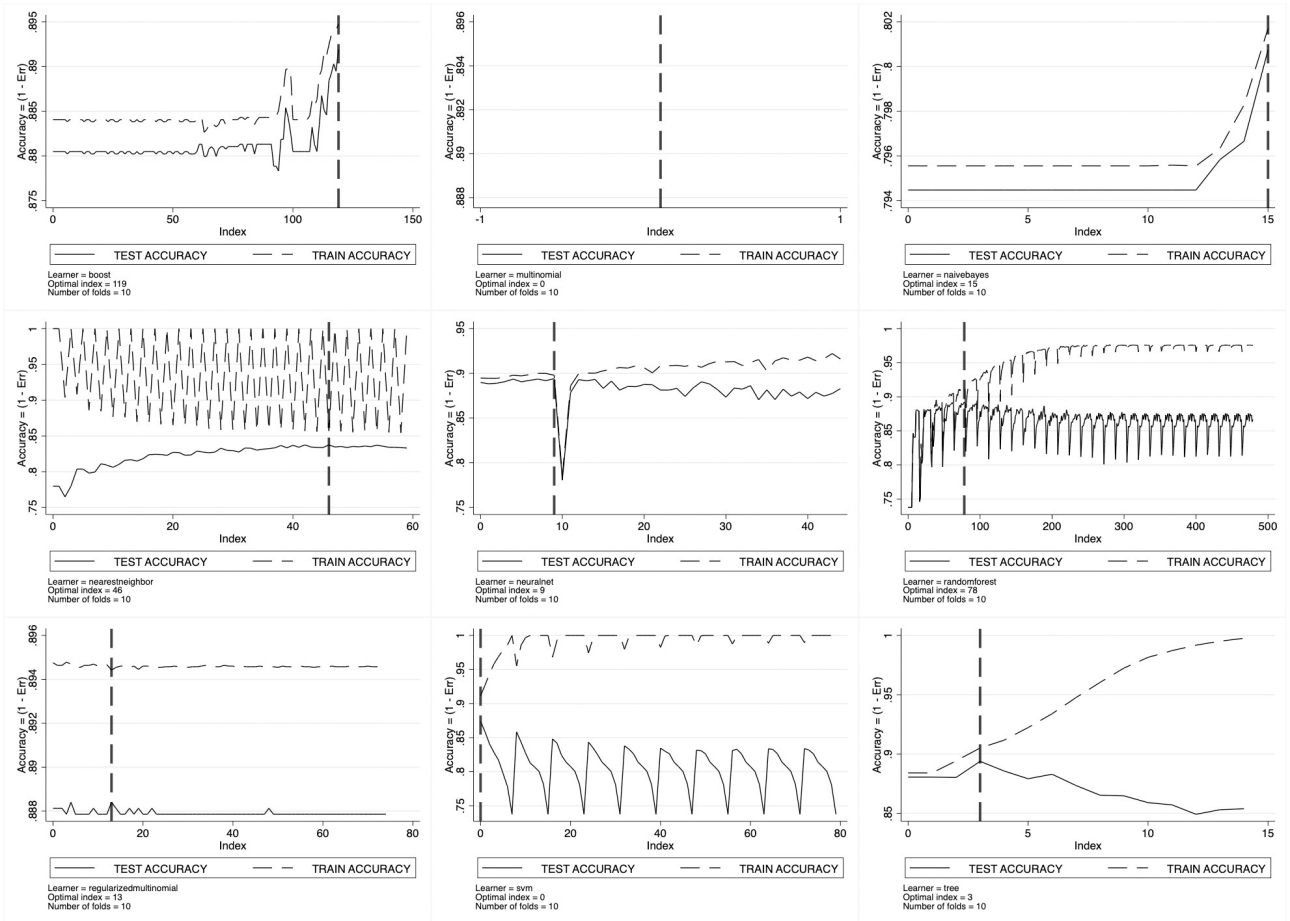
Learners’ optimal tuning using 10-fold cross-validation for PE.

**Fig 5:**
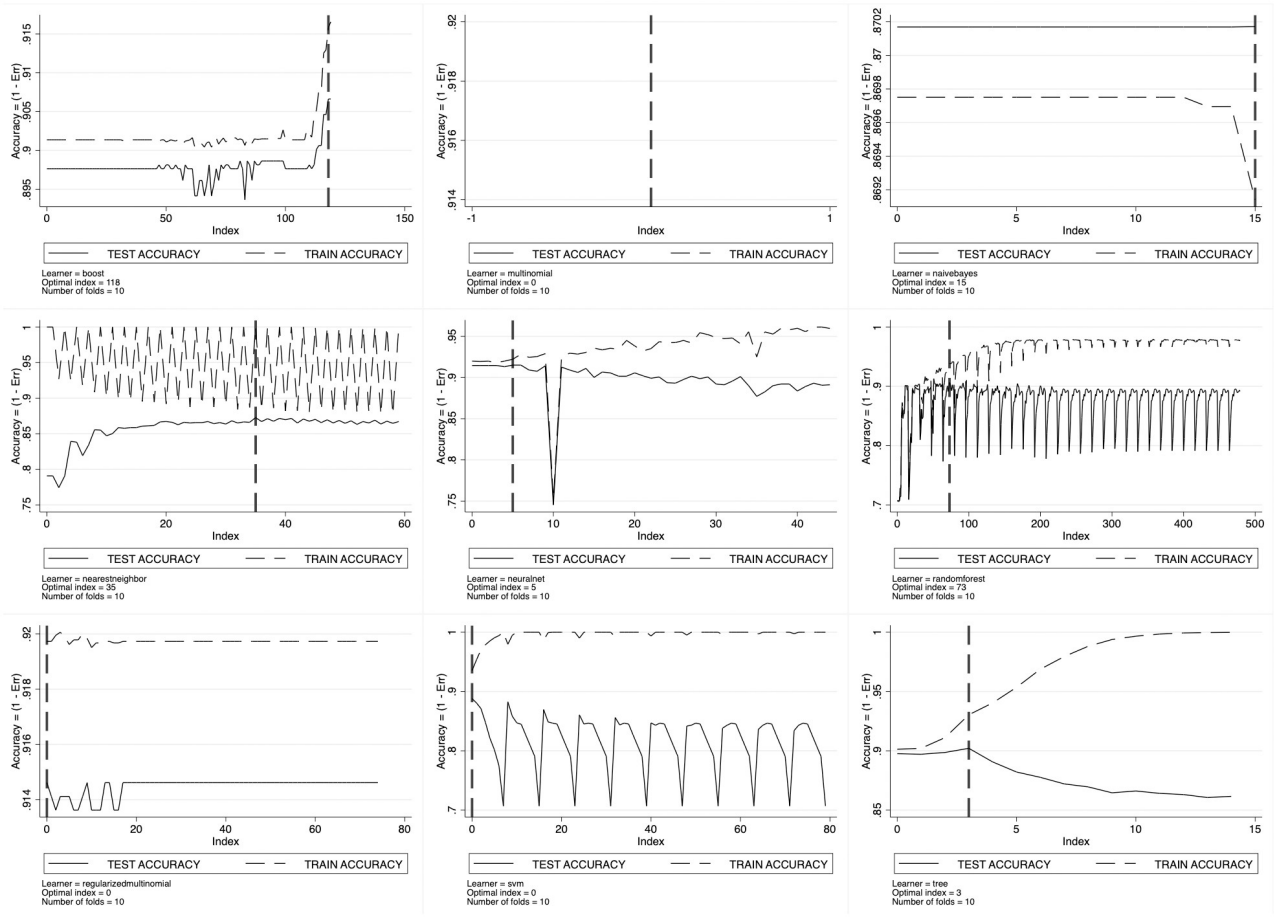
Learners’ optimal tuning using 10-fold cross-validation for LS.

For every considered machine learning algorithm, cross-validation results are set out in Fig 3 for SSH, Fig 4 for PE and Fig 5 for LS. Here, we observe the pattern of the training- and the test-accuracy by ERC domain as a function of the grid index, with each point of the grid representing a specific configuration of the tuning parameters. As long as the grid index increases, we have that all the tuning parameters increase thus entailing a trade-off between prediction bias and variance. As expected, the training-accuracy sets out a monotonic increasing pattern going asymptotically to one. This is the well-known *overfitting* phenomenon characterizing in-sample prediction accuracy. To avoid overfitting, ML scholars suggest looking at the test-accuracy instead. The test-accuracy—generated by cross-validation—represents the correct out-of-sample accuracy to look at, and its maximand is the optimal tuning parameters’ configuration.

Once the optimal tuning parameters’ configuration is found, we use it to predict the probability of link formation. Table 2 sets out point accuracy results for SSH.

**Table 2:** Point estimation of the training- and of the test-accuracy in predicting network link formation by learner. ERC domain: SSH.

	TRAIN ACCURACY	TEST ACCURACY
Tree	.925515	.9180858
Support vector machine	.9436323	.9090089
Regularized multinomial	.9257615	.9251406
Random forest	.9506328	.9269076
Neural network	.9346718	.9286702
Nearest neighbor	1	.8729601
Naive Bayes	.9028075	.8984187
Mutinomial	.9258791	.9248887
Boosting	.924507	.9203522

Looking at the column reporting test-accuracy, it is immediate to see that for the SSH sector every learner fits well our network data considering all exogenous and endogenous variables, with Neural Network obtaining the best accuracy performance (92.9%). Except for the Nearest neighbor (87%) and Naive Bayes (89%), all the other learners reach accuracy values higher than 90%. For the PE domain, the Neural Network classifier achieves an accuracy of 89.4% (see Table 3).

**Table 3:** Point estimation of the training- and of the test-accuracy in predicting network link formation by learner. ERC domain: PE.

	TRAIN ACCURACY	TEST ACCURACY
Tree	.9050687	.893847
Support vector machine	.911601	.8739745
Regularized multinomial	.8944232	.8884026
Random forest	.9157441	.8924846
Neural network	.8979616	.8941187
Nearest neighbor	.8570737	.8375163
Naive Bayes	.8016995	.8007404
Mutinomial	.8945442	.8878584
Boosting	.8947559	.8919418

Finally, for the LS domain, the best accuracy performance was achieved again by the Neural Network learner (91.5%) (see Table 4).

**Table 4:** Point estimation of the training- and of the test-accuracy in predicting network link formation by learner. ERC domain: LS.

	TRAIN ACCURACY	TEST ACCURACY
Tree	.9303819	.9021244
Support vector machine	.93543	.8881393
Regularized multinomial	.9197313	.9146194
Random forest	.9380926	.9066119
Neural network	.9221718	.9151169
Nearest neighbor	1	.8726791
Naive Bayes	.8691407	.8701717
Mutinomial	.9197312	.9146169
Boosting	.9158487	.9066269

**Fig 6:**
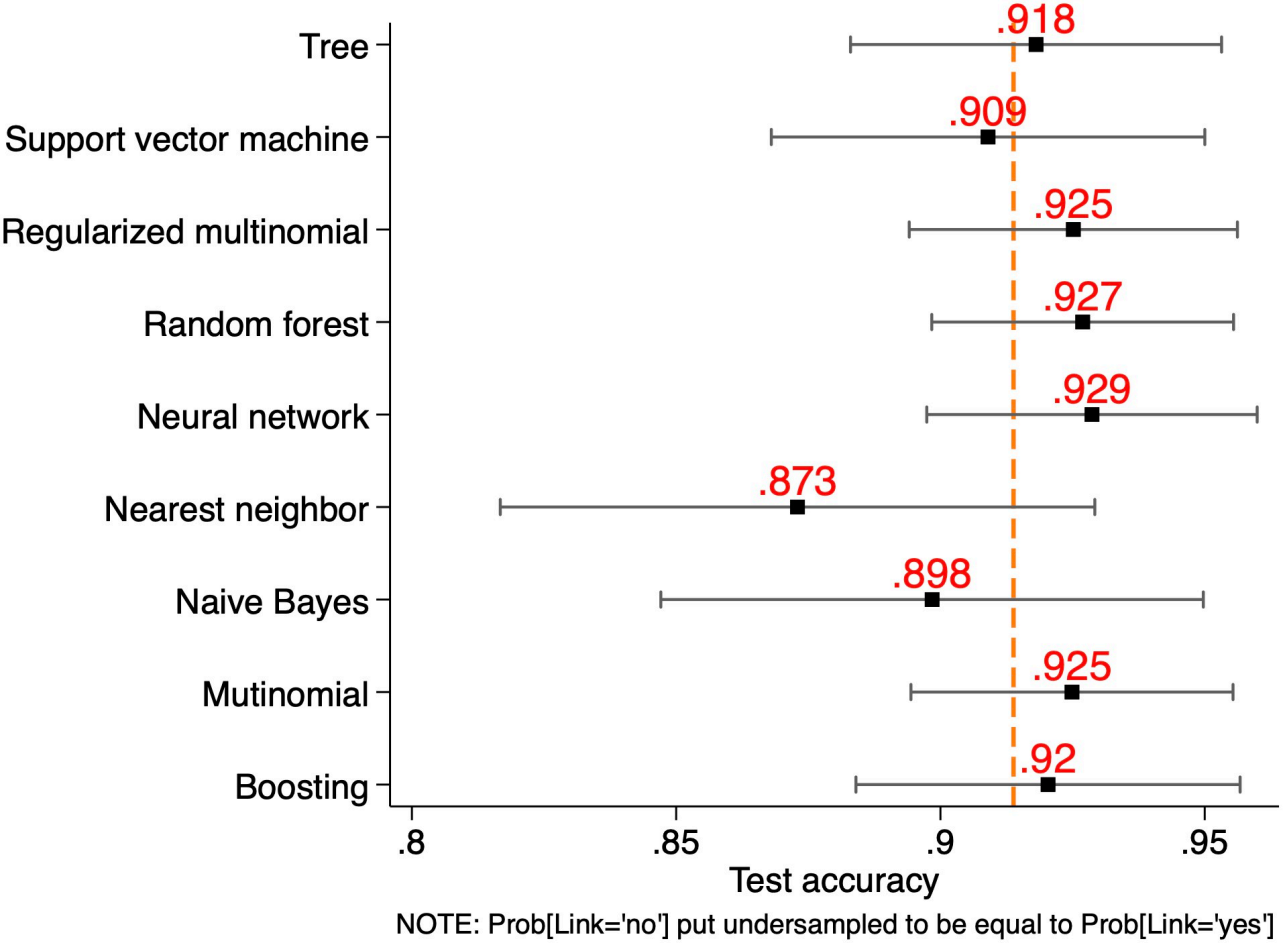
Point and interval prediction accuracy performance by learner for SSH.

**Fig 7:**
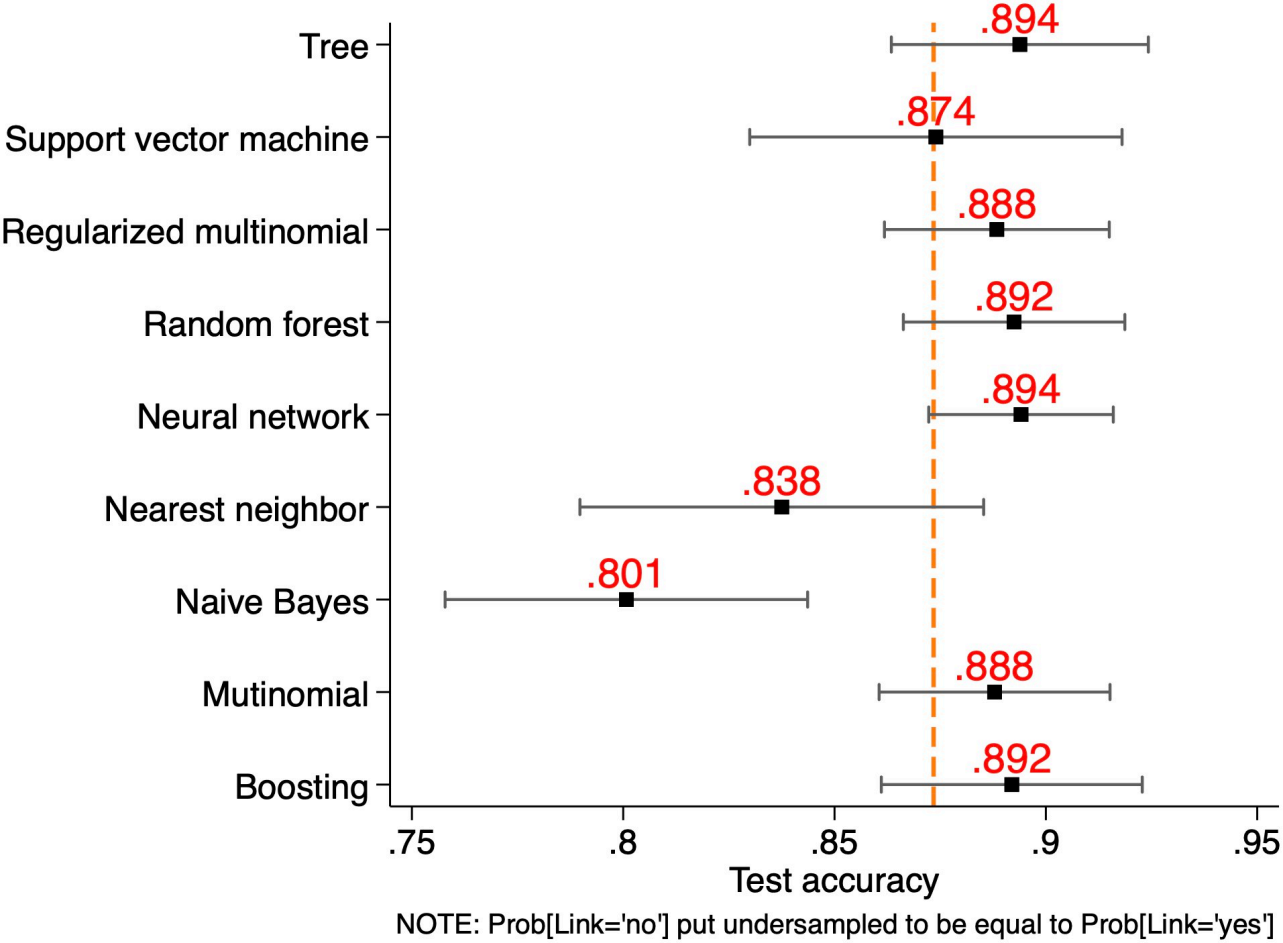
Point and interval prediction accuracy performance by learner for PE.

**Fig 8:**
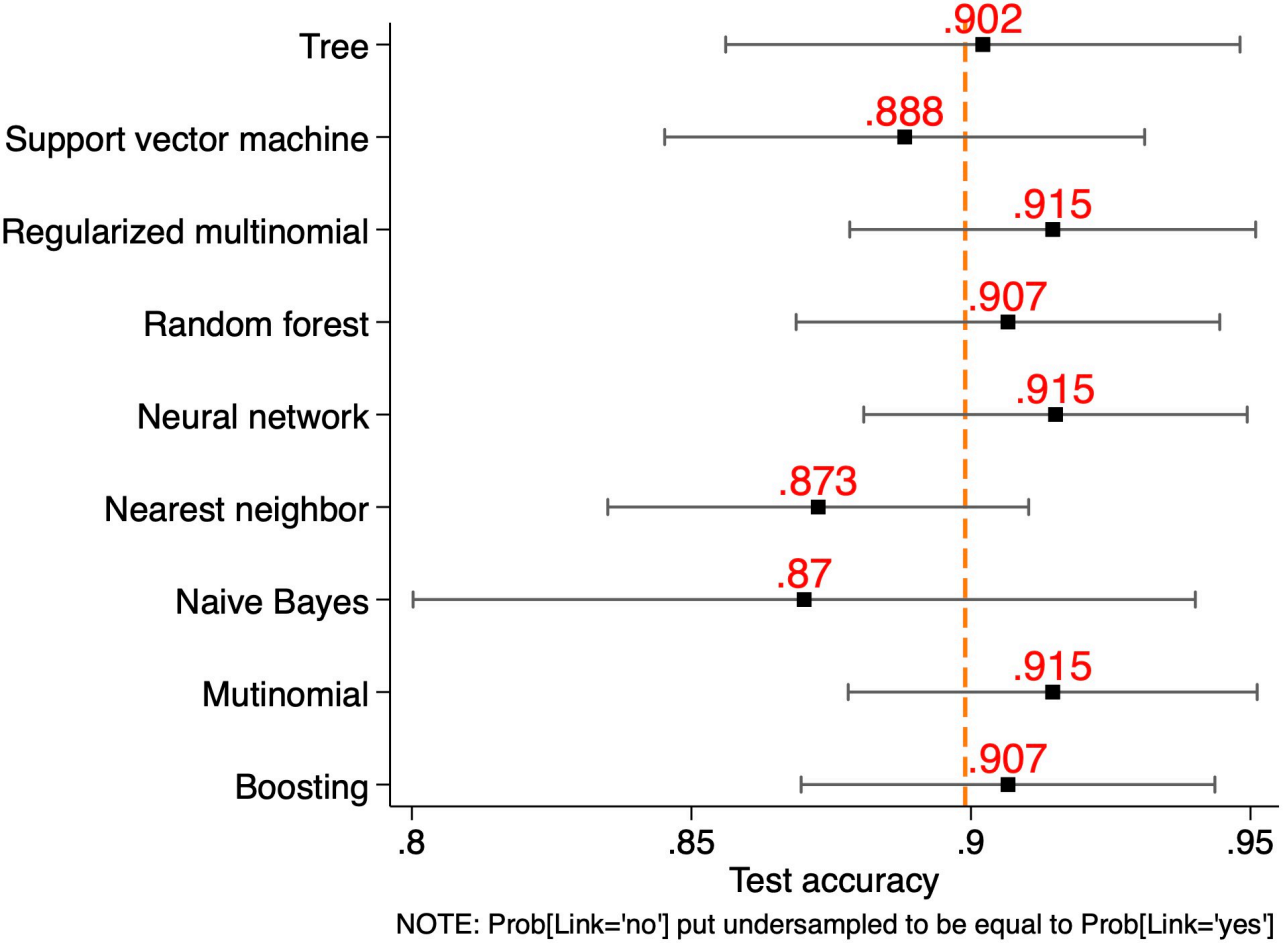
Point and interval prediction accuracy performance by learner for LS.

As well-known, assessing a learner’s predictive performance just on the basis of its point average accuracy may be misleading, as the estimation of such accuracy may be in some cases severely imprecise. For this reason, it is appropriate to jointly consider accuracy mean (as done above) and standard deviation, thus obtaining an accuracy’s confidence interval. This is reported in Fig 6 for SSH, Fig 7 for PE and Fig 8 for LS, where our learners are compared not only over their average accuracy but also by taking into account estimation precision. Interval estimation at 95% significance level shows that the predictive precision of our learners is rather uneven compared to point estimation. For the different ERC domains the best performers in terms of interval estimation are Random Forest (SSH) and Neural Networks (PE and LS), all having a tighter distribution around their accuracy mean when compared to the other learners.

Previous results hinge on a model specification including both exogenous and endogenous features. As stressed earlier in this paper, however, a model embedding node endogenous features can only predict link probabilities for nodes that already belong to the extant network. If we are interested in investigating how brand-new links are formed considering nodes that were not previously part of the network, we have to rely on a model specification incorporating only exogenous features. Accuracy results regarding such a model are visible in Table 5 for SSH, Table 6 for PE, and Table 7 for LS, where it is immediate to recognize a general reduction in prediction accuracy for all the learners considered. Considering only exogenous variables for SSH (Table 5) the best accuracy performances are given by the Support vector machine (63.9%) and the Neural Network (63.4%).

**Table 5:** Point estimation of the training- and of the test-accuracy in predicting network link formation by learner. Only exogenous features considered. ERC domain: SSH.

	TRAIN ACCURACY	TEST ACCURACY
Tree	.6632826	.6321011
Support vector machine	.7409018	.6393574
Regularized multinomial	.6265143	.6137518
Random forest	.6690841	.6309015
Neural network	.6747776	.634275
Nearest neighbor	.687374	.6279877
Naive Bayes	.6084446	.5977711
Mutinomial	.6266755	.613509
Boosting	.6411785	.6178417

**Table 6:** Point estimation of the training- and of the test-accuracy in predicting network link formation by learner. Only exogenous features considered. ERC domain: PE.

	TRAIN ACCURACY	TEST ACCURACY
Tree	.7155144	.702152
Support vector machine	.8480048	.7097012
Regularized multinomial	.7164662	.7120321
Random forest	.7206208	.723199
Neural network	.7261605	.7135913
Nearest neighbor	1	.7086576
Naive Bayes	.7126867	.7130731
Mutinomial	.7160335	.7125517
Boosting	.7190917	.7138463

**Table 7:** Point estimation of the training- and of the test-accuracy in predicting network link formation by learner. Only exogenous features considered. ERC domain: LS.

	TRAIN ACCURACY	TEST ACCURACY
Tree	.6835182	.6384825
Support vector machine	.9885519	.6778985
Regularized multinomial	.6950736	.6712026
Random forest	.6834646	.6774247
Neural network	.6839997	.6745402
Nearest neighbor	.7161513	.6625534
Naive Bayes	.4677673	.4435177
Mutinomial	.6952876	.6716857
Boosting	.6826616	.6817517

For the PE sector, we obtain an accuracy of 72.3% (Random forest) when we treat only exogenous features (Table 6).

For the LS sector, with only the exogenous variables in the model, the best performance is given by Boosting (68.1%) (Table 7).

One of the objectives of this study was to evaluate the relative importance of endogenous features in predicting the links in joint projects. The findings have revealed that, by excluding the two network endogenous components, we lose approximately between 20% (PE and LS) and 30% (SSH) of accuracy. A non-trivial result that enhances and emphasizes the role of network configurations in link prediction. This finding stresses the trade-off we were referring to in the section titled “Problem formulation” entailing that, if we want to know more about the evolution of a network structure by also incorporating newcomer nodes, we have to pay a cost in terms of reduced prediction accuracy that in our case may be sizable.

### Feature partial effects for link formation

In this section, we provide evidence about the role played by every single feature viewed as a driver of the probability to create a link. In machine learning, feature importance measures are generally obtained by computing the contribution of each feature to increase the test-accuracy of a learner (or, equivalently, to reduce the test-error). This approach is no doubt informative, but lacks an essential element, i.e. understanding which is the contribution of every single feature to the probability to produce a link. This has to do with the marginal effect (or partial derivative) of a feature on the link probability. As supervised learning is statistically equivalent to estimating the conditional mean of the target *y* given the vector of features *X*, defining and computing an average partial (or marginal) effect (APE) is straightforward, with the formula taking on this form:

$$\begin{array}{c} \text{APE}\left(y,x_{j}\right)=\frac{\partial E(y|x_{j},{\bar{X}}_{-j})}{\partial x_{j}}=\frac{\partial Prob(y=1|x_{j},{\bar{X}}_{-j})}{\partial x_{j}} \end{array}$$ (6)

where ${\bar{X}}_{-j}$ indicates all the features different from *x*_*j*_ evaluated at their sample mean. Observe that the second equality of Eq (6) derives from the fact that *y* is binary.

Eq (6) can be easily estimated on the sample, and a simple plot of $\hat{E}\left(y|x_{j},{\bar{X}}_{-j}\right)$ is sufficient to identify the curvature of its derivative over *x*_*j*_. In this way, we can derive both the shape of the probability to generate a link as a function of every *x*_*j*_ given that all the other features are held fixed at their mean, and the sign and size of the derivative. In practice, this allows us to gauge whether the link probability increases (decreases) as *x*_*j*_ increases (decreases) and to what amount, and if there are different patterns in different regions of the support of *x*_*j*_. We consider for this analysis the complete model specification (i.e., both network exogenous and endogenous features).

The APE estimation can be carried out by every single learner, but results may be characterized by a large variability. In other to reduce estimation variance, we opt to rely on an aggregation (average value) of the derivatives obtained learner-by-learner. Aggregating learners’ estimates entails to estimate a “super-learner” generally having smaller variance at reasonable costs in terms of reduced point estimate precision (bias).

**Fig 9:**
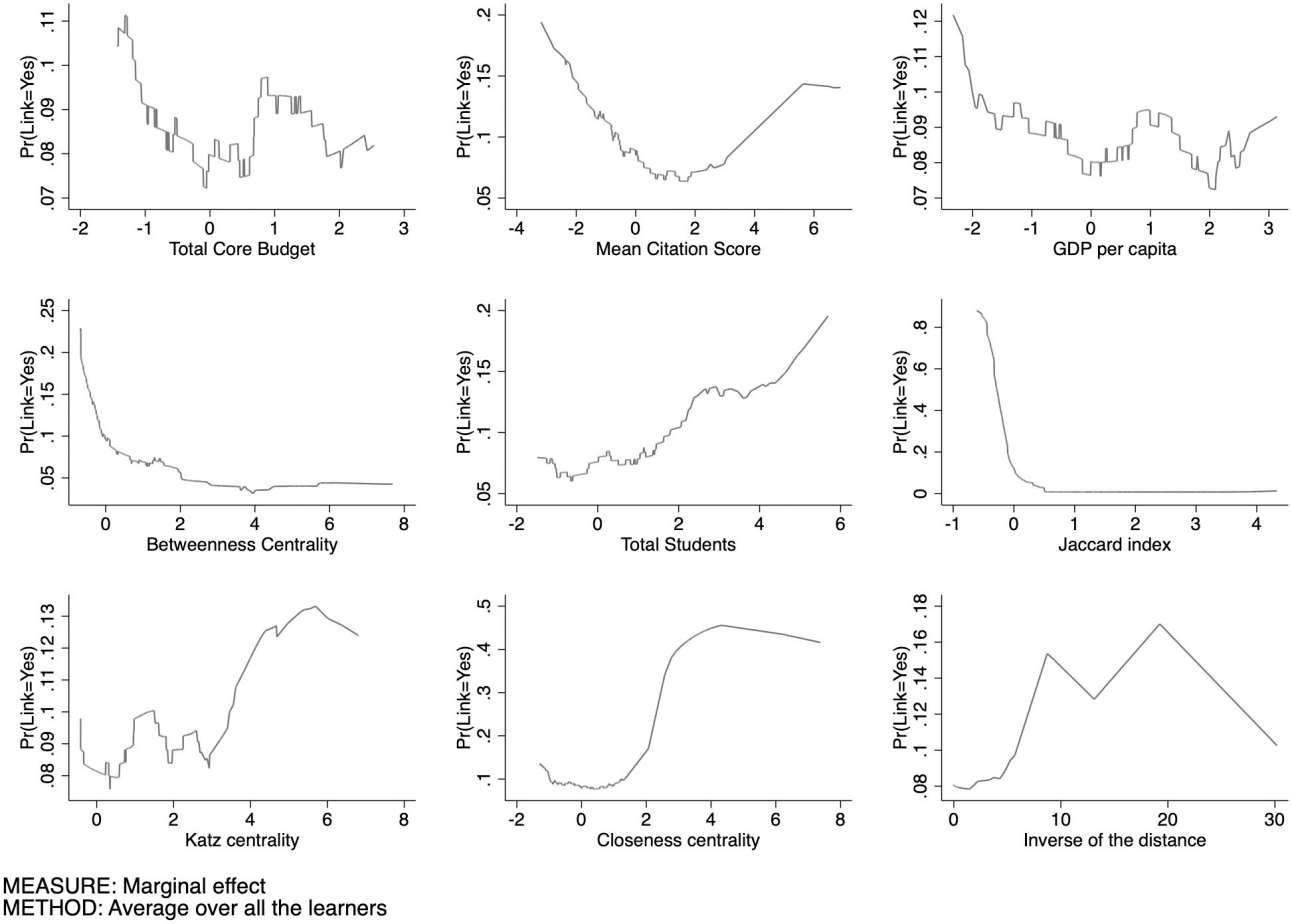
Conditional partial expectation of the probability to have a link for SSH.

The first domain under consideration is the SSH. Fig 9 sets out our estimates of $Prob(y=1|x_{j},{\bar{X}}_{-j})$ for the SSH sector, that is, the conditional partial expectation of the probability to have a link as a function of every single feature. Observe that, in order to aggregate the features of the different nodes, we use their arithmetic mean.

**Fig 10:**
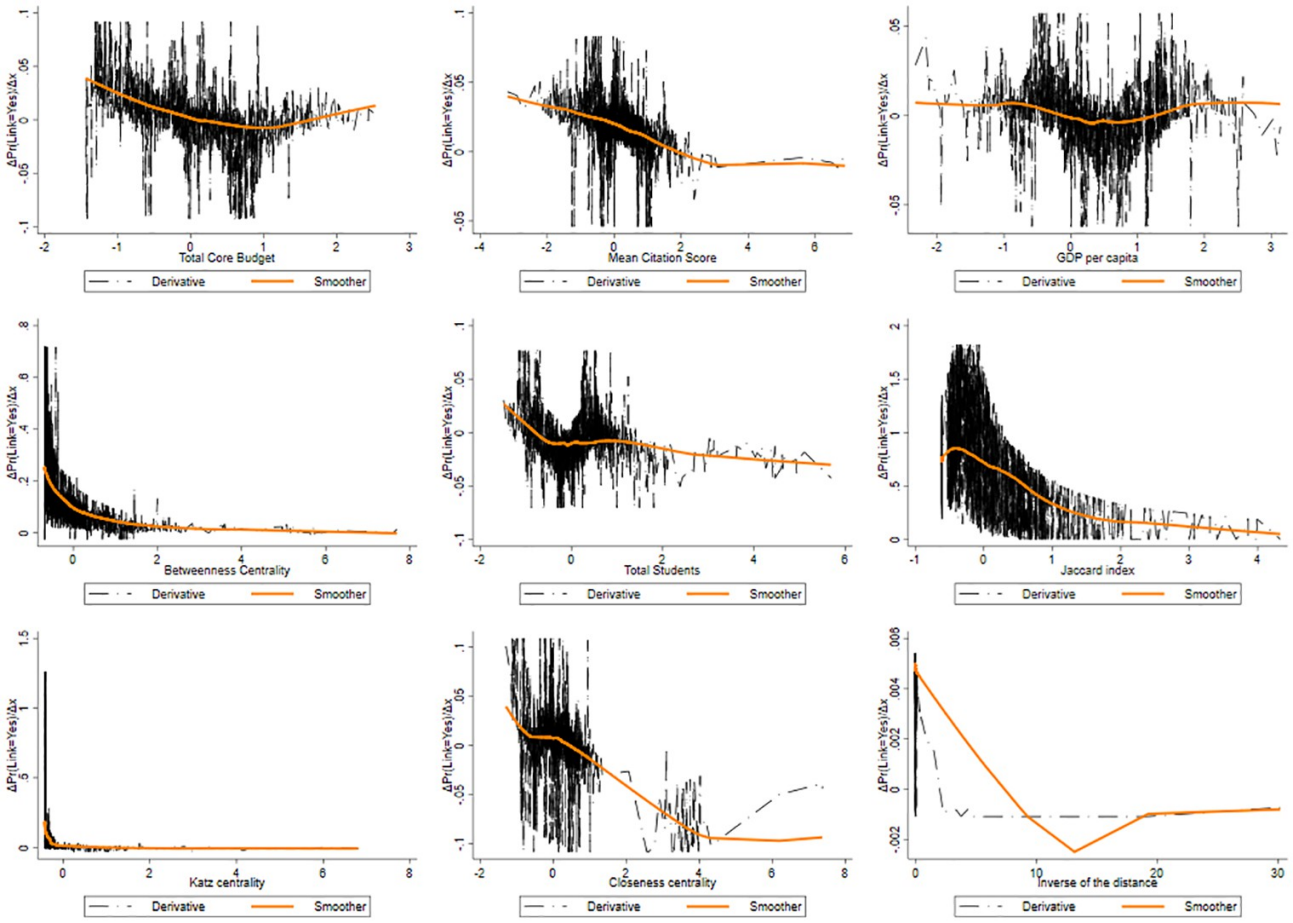
Pattern of the derivative of the probability of produce a link as a function of the features for SSH.

The total core budget exhibits a decreasing pattern of $Prob(y=1|x_{j},{\bar{X}}_{-j})$ followed by a stabilization phenomenon, as showed by Fig 10 where the APEs are plotted. We can summarize this finding as follows: initially, if either (or both) universities have a low core funding, it is more likely that they will collaborate in publicly funded research projects. However, as the core funding increases beyond a certain threshold, this probability decreases. This suggests a non-linear relationship between core funding and collaboration probability, where lower levels of core funding foster collaboration, but as the funding increases over a certain threshold, the motivation for cooperation declines.

The mean citation score presents a U-shaped form: at low level of this index, universities exhibit a high level of APE, that however decreases dramatically near the average of this feature, to then growing towards higher values. Universities having a lower scientific reputation tend to connect together, as well as universities with a higher reputation tend to be more prone to create a link. It is plausible to conclude that organizations tend to choose partners that are similar with respect to their scientific reputation. In this context, the U-shaped pattern indicates a similarity effect.

Universities located in more economically developed regions tend to have a lower probability to lay links. When comparing the findings related to low core funding and low GDP per capita, both demonstrate a similar trend in terms of collaboration probability in publicly funded research projects. In both cases, higher levels of core funding or GDP per capita are associated with a lower likelihood of universities engaging in joint projects. There is evidence of dissimilarity effects in inter-university collaboration, emphasising the importance of bridging regional disparities and fostering collaboration as a means to enhance research and innovation across the EU (these are to be taken however as mere speculations, as we are unable to test such assumptions in this paper). The lower capacity of universities in poorer regions to secure funding, as well as their relatively limited availability of skills and resources, can contribute to the observed dissimilarity effects in inter-university collaboration.

The pattern exhibited by the Betweenness centrality displays a symmetric J-shaped form. At lower level of the Betweenness, the $Prob(y=1|x_{j},{\bar{X}}_{-j})$ is higher. We then observe, again, a strong drop at relatively high levels, leading to a long plateau at a higher level of Betweenness where it reaches its minimum. This pattern suggests that when universities are poorly central in the network (low Betweenness), i.e. its mediating role is negligible, small increases in its centrality reduces the probability to generate links and, possibly, larger centrality. This effect gets to an end rather soon and fades away along the plateau, until the probability of new links gets locked in. Those nodes with higher Betweenness centrality often connect nodes found in different communities, and usually they are far from each other.

As for university size, we observe a quite clear pattern, as it seems to have a positive correlation with the link formation. The probability of activating a link increases significantly when universities get larger.

The pattern of the Jaccard coefficient is similar to that of the Betweenness centrality; these two features move in the same direction. There may be complementarity effect in this pattern, as well as for the Betweenness centrality. Complementarity is a crucial aspect in collaborations, and is explained by the resource-based view of strategic alliances. Two universities with common neigbours are likelier to share the same scientific cluster, and probably have similar competences. Pairs of universities with a higher Jaccard coefficient have a greater cognitive proximity that can reduce the likelihood of receiving funding, in favor of a diversification of the scientific competences. This result suggests that universities tend to create links with non-neighboring universities (perhaps in different communities). Based on our experiments, we observed that the Jaccard coefficient is a largely effective feature for link prediction. We can observe, compared to the other features, that the range of probability is much higher (from 0 to 0.85). In the context of predicting links between universities in the Horizon 2020 programme, a higher Katz centrality score for a university implies that it has stronger connections and influence within the network. Therefore, universities with higher Katz centrality scores are more likely to have links or collaborations with other universities within their clusters or communities. This analysis can provide valuable information for policymakers, it can aid in identifying universities that may benefit from targeted support and resources to further strengthen their connections and collaborations within the network. The “S” trend observed in Closeness centrality suggests that certain universities within the network exhibit varying degrees of proximity to other nodes. The Closeness centrality scores are relatively low in the start of the trend, indicating that certain universities are quite distant and have a tendency to link less. As the trend progresses, the Closeness centrality scores increase, signifying that the universities become progressively more connected and accessible to others. It highlights the emergence of key universities that act as information brokers, facilitating efficient communication and information diffusion within the network. Finally, the inverse of the distance (proximity) exhibits an oscillating pattern with an initial phase showing a low probability of creating a link between universities that are geographically distant. This is followed by a subsequent increase, then a slight decrease, another rise, and a subsequent descent as the proximity between the universities increases. Overall, understanding the relationship between geographic distance and the probability of link formation provides valuable insights into the dynamics of collaboration in research networks. It highlights the importance of proximity in fostering collaborations, while also acknowledging that other factors beyond distance may influence the likelihood of link formation between universities. Furthermore, it is crucial to consider the nature of the network under analysis, especially in the case of international programmes like Horizon 2020, which emphasize collaborations among institutions from different countries.

**Fig 11:**
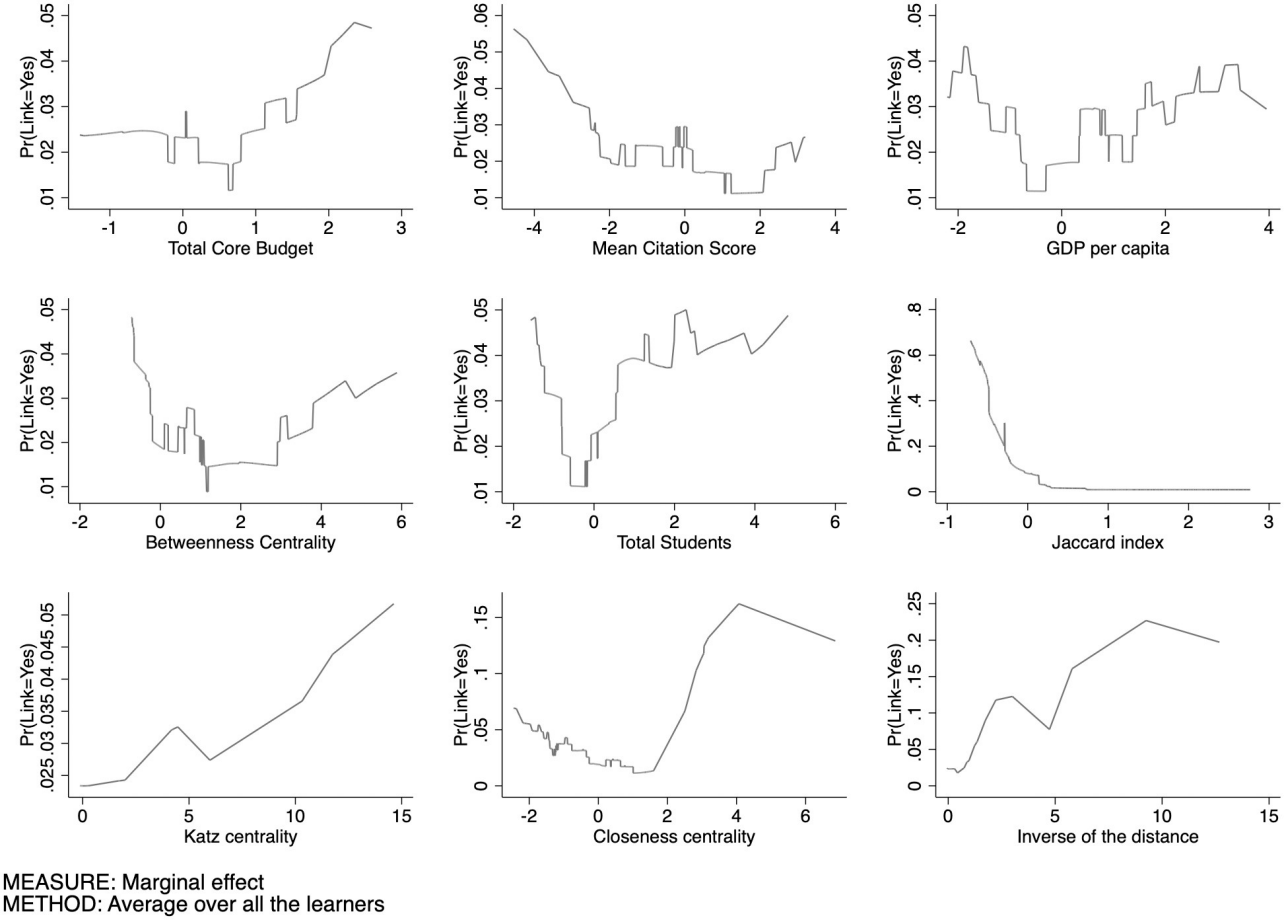
Conditional partial expectation of the probability to have a link for PE.

**Fig 12:**
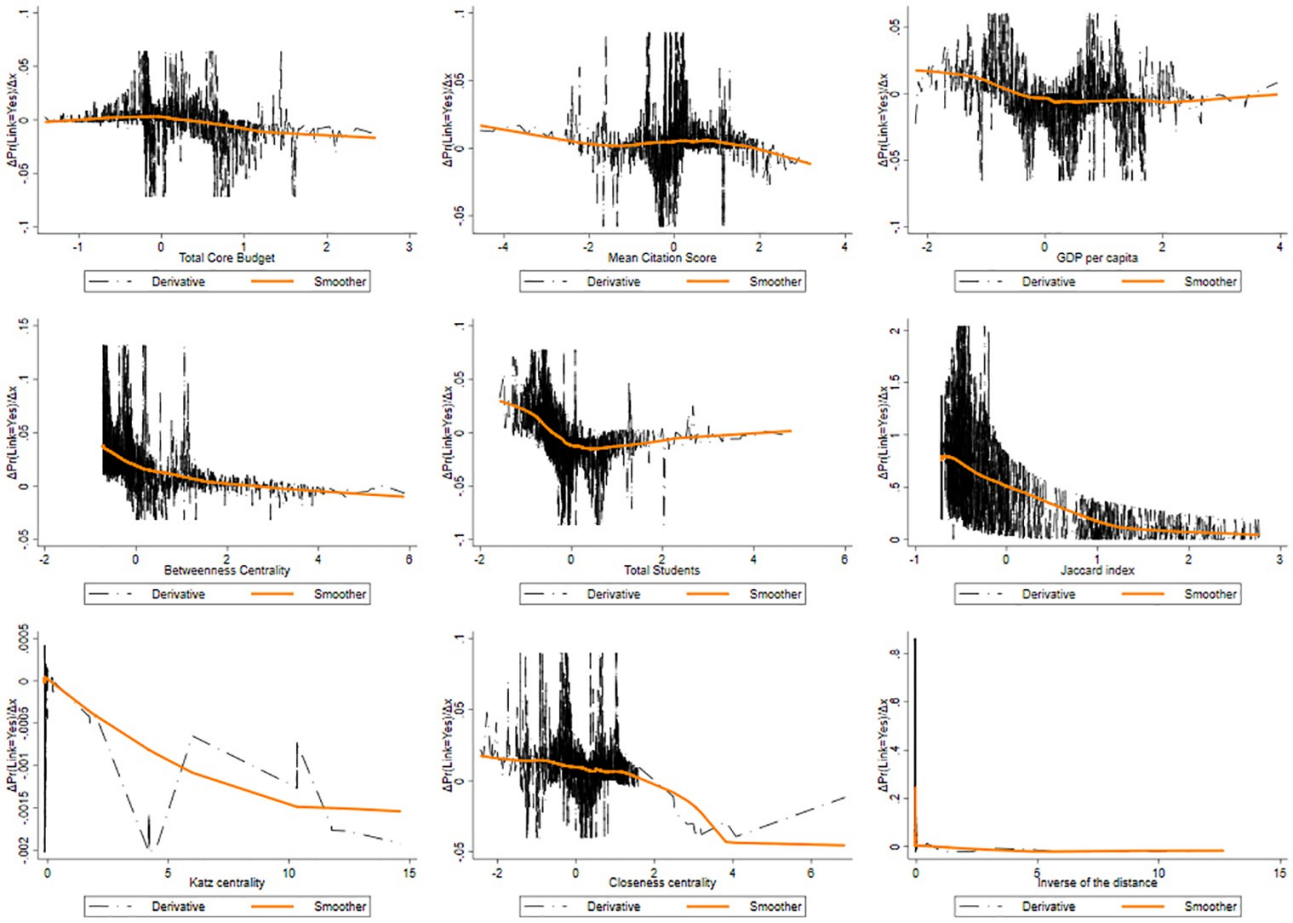
Pattern of the derivative of the probability of produce a link as a function of the features for PE.

For Physical and Engineering Sciences (Fig 11) the pattern shows a lower predicting responsiveness than in SSH, highlighting the limits of using the exogenous variables in the link prediction. Furthermore, we notice here the presence of a greater prediction uncertainty than in the SSH domain. APEs results for PE are showed by Fig 12.

PE, the general pattern of the total core budget displays a plateau in the initial half, indicating that the starting range has the lowest incidence of link creation. The examination of the entire core budget shows an upward tendency in the second section of the distribution. The citation score variable shows a lower probability to form links in second half. Universities in areas with a lower GDP per-capita are likelier to collaborate in publicly funded research projects, after which the tendency begins to decline and ultimately stabilizes. Universities with lower initial Betweenness centrality scores interact less. These couples are more likely to cooperate as the Betweenness centrality grows. In PE network, we show a lower predictive responsiveness as compared to SSH findings. The trend of university size is increasing around the zero point. It is interesting to note, instead, that the Jaccard coefficient shows a very similar trend to that of the SSH sector. Katz centrality reveals a consistent increasing pattern, with a low predictive responsiveness. The “S” pattern in Closeness centrality indicates behavior comparable to the SSH network. The analysis of the inverse of the distance reveals an increasing pattern. It quantifies the degree of proximity or closeness between universities, with higher values indicating closer proximity. Universities that are closer in terms of geographic distance exhibit a higher probability to collaborate.

**Fig 13:**
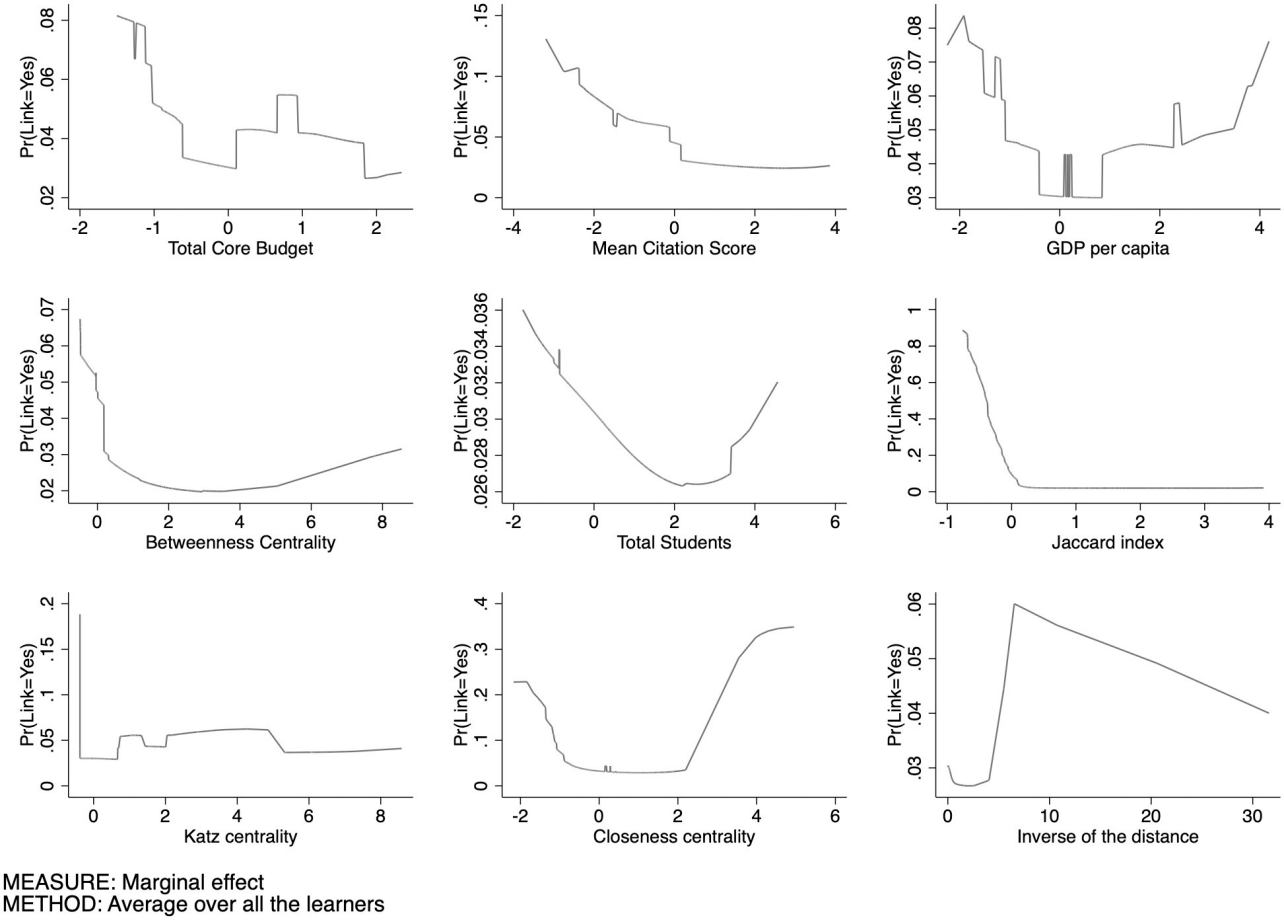
Conditional partial expectation of the probability to have a link for LS.

As for the link prediction in Life Sciences (Fig 13), we observe a decreasing trend in the influence of the total core budget on the probability of collaboration between two universities. This suggests that as the total core budget increases, the likelihood of collaboration between universities decreases. The mean citation score trend is decreasing, with a flattening of the line in the higher values of the variable. GDP per-capita also exhibits a U-shaped curve describing the sub-population of universities contributing to link prediction. There would seem to be a similarity effect between universities regarding the GDP of the regions in which they are located. Universities located in areas with a lower GDP tend to link up more; similarly, universities located in areas with a higher GDP link together. The bridging role of the university on the link creation loses its initial potential with higher values of Betweenness centrality. Our analysis reveals a U-shaped relationship between the size of universities and the probability of collaboration. This U-shaped pattern suggests that there is some degree of similarity in terms of university size that promotes collaboration between universities. However, we observe a low level of responsiveness. This means that the probability of collaboration between universities is not highly sensitive or responsive to changes in university size. The Jaccard index shows similar values to the SSH and PE domains. Moreover, when two universities have low Katz scores, there is an initial high probability of collaboration, followed by an immediate plateau in the probability. We also note a low level of responsiveness of this variable in determining collaboration probabilities. The Closeness centrality exhibits a U-shaped pattern. This finding suggests that nodes with similar values of Closeness centrality in the network tend to exhibit a higher propensity for connecting with each other compared to nodes with intermediate values. Understanding the propensity for nodes with similar Closeness centrality values to connect can provide insights into the underlying dynamics of the network. It suggests that there are certain attractor mechanisms or clustering tendencies that operate based on Closeness centrality values. The inverse of the distance, which captures the geographic proximity between universities, exhibits a trend characterized by low levels of probability for distant universities, followed by an increase in probability up to a certain threshold (around the value of 10), and then a slow decrease.

**Fig 14:**
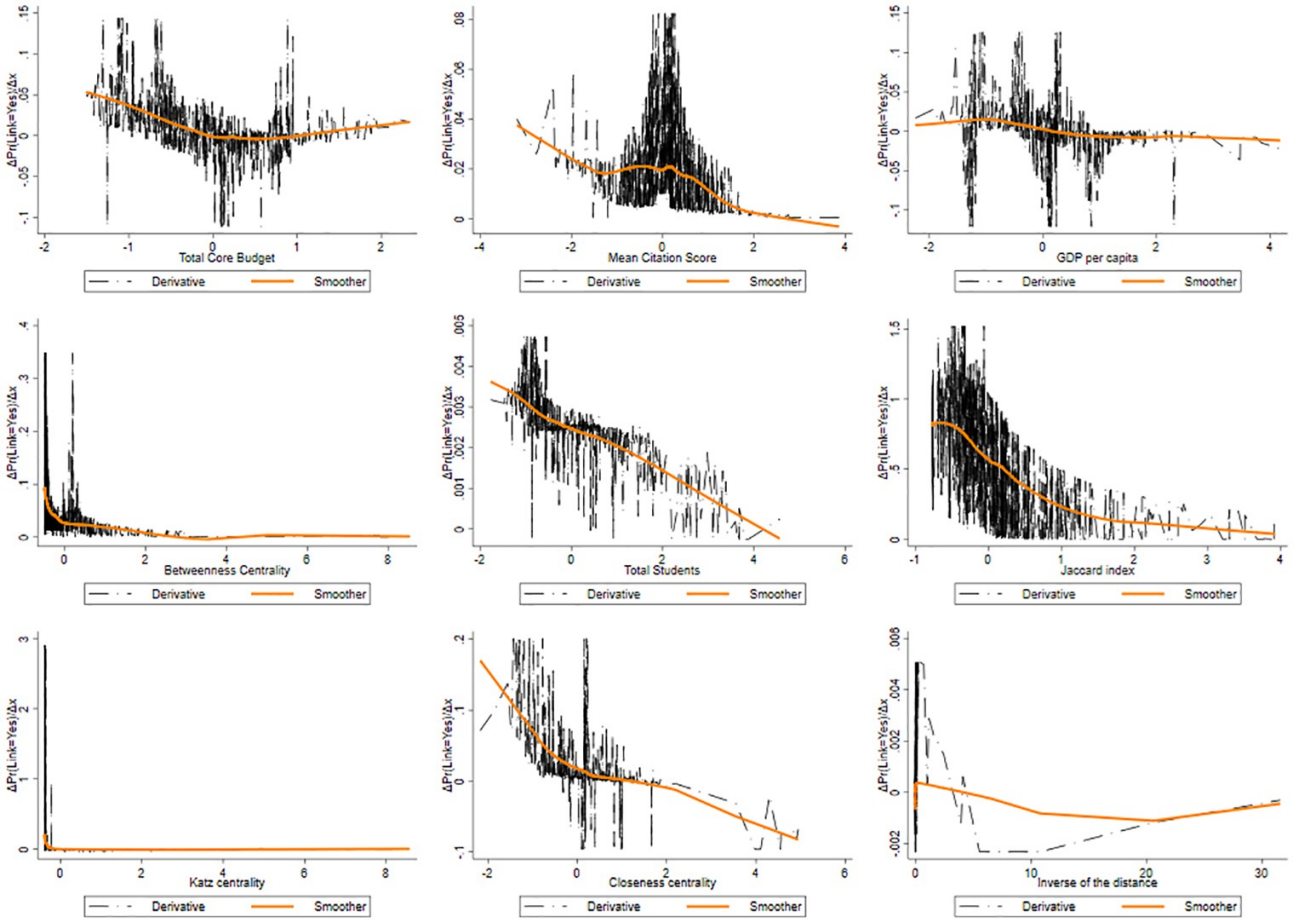
Pattern of the derivative of the probability of produce a link as a function of the features for LS.

As in the other domains, the Jaccard coefficient has the highest impact on link prediction. Above a certain threshold limit, the probability is steady and closer to zero. The effect of common neighbors in creating connections tends to become saturated in terms of community capacity to offer additional skills. APEs results for LS are showed by Fig 14.

In order to explain the differences emerged between the PE and LS domains compared to the SSH domain in our results, we can refer to the major complexity of the types of organizations and institutional collaborations operating within the projects taking place within the PE and LS domains. These consist of collaborations that often include private companies, of organizations operating through large R&D laboratories that carry out more explorative and risky research, characterized by larger irreversibility and huger sunk costs.

**Fig 15:**
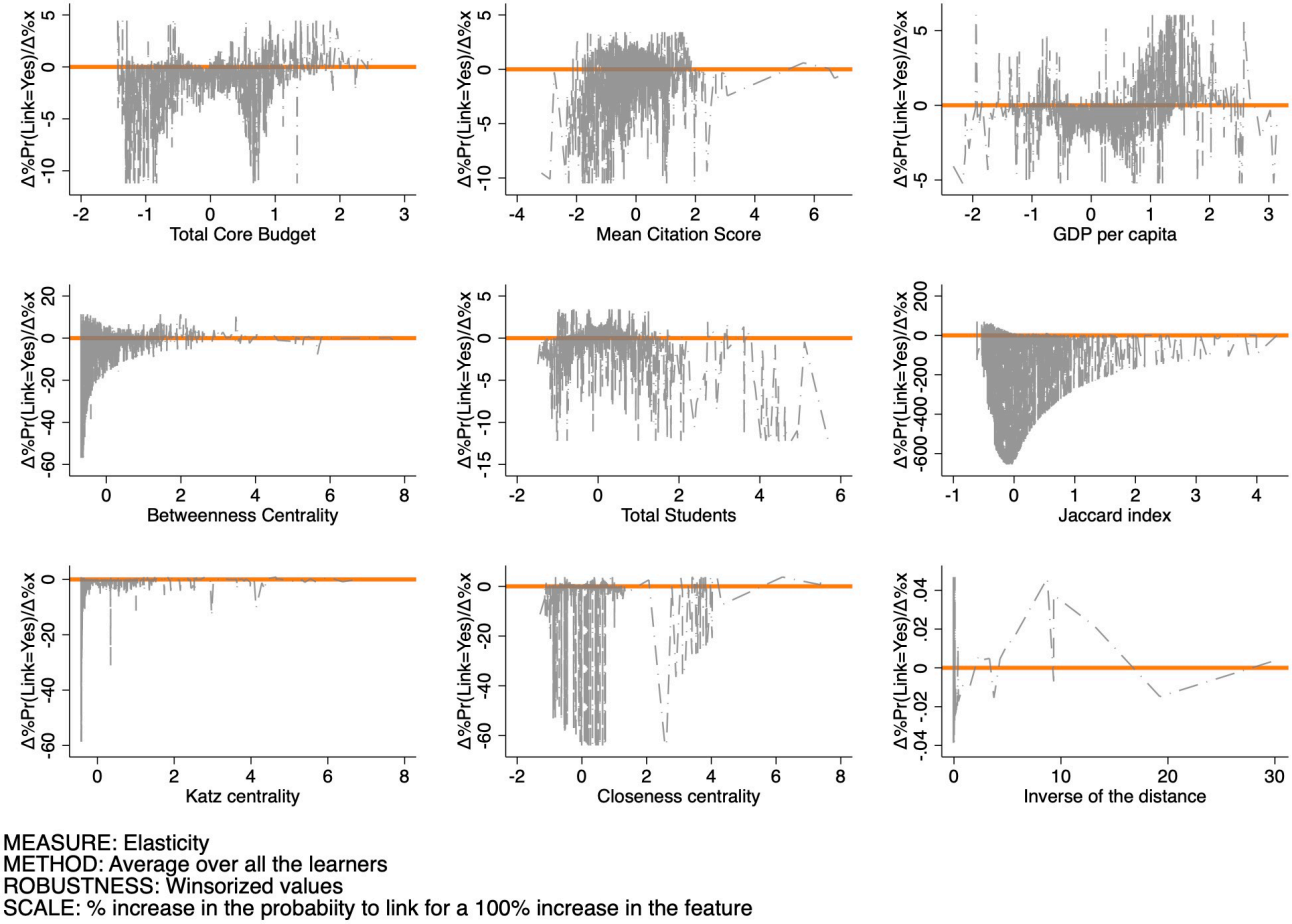
Pattern of the elasticity of the probability of produce a link as a function of the features for SSH.

**Fig 16:**
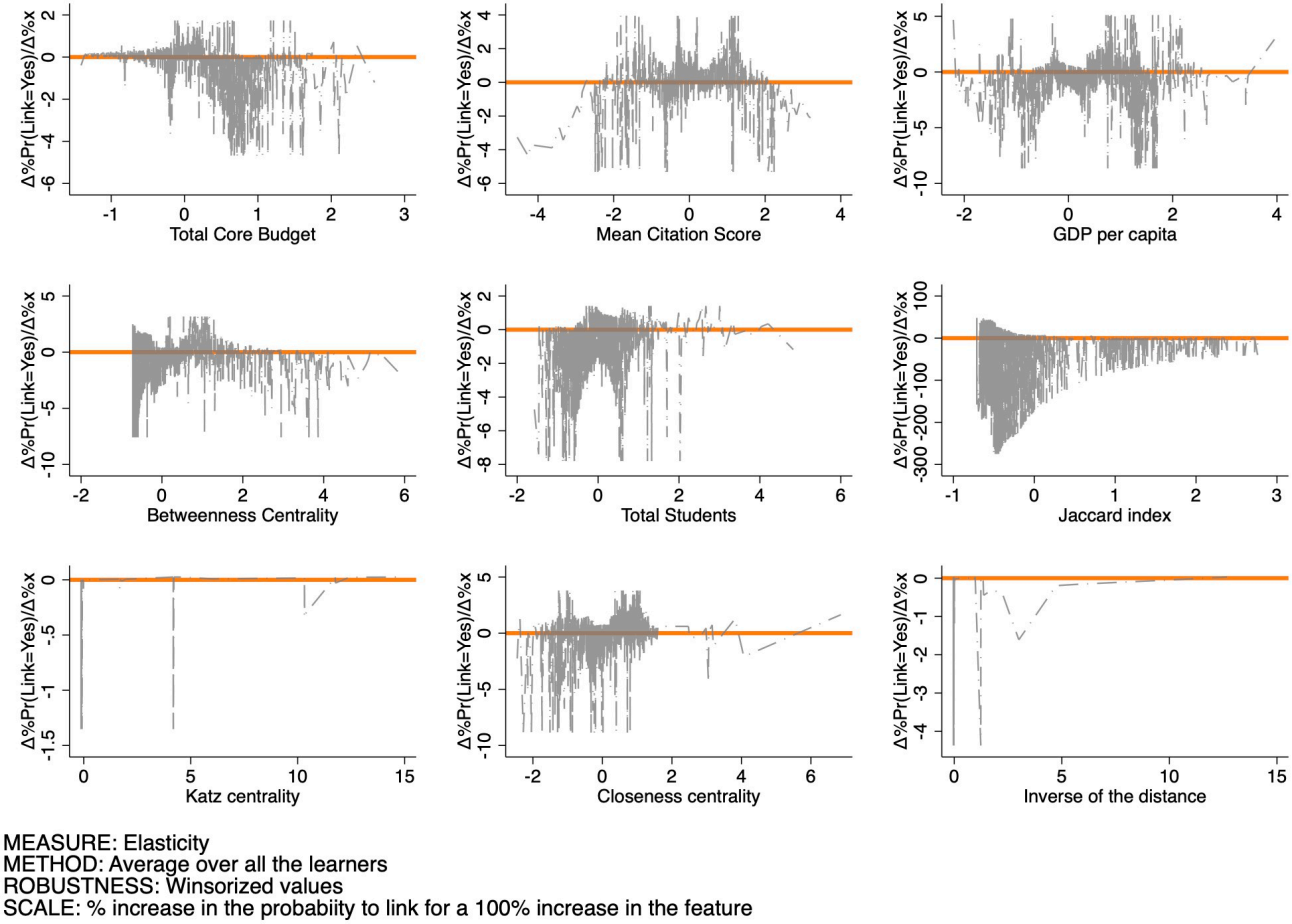
Pattern of the elasticity of the probability of produce a link as a function of the features for PE.

**Fig 17:**
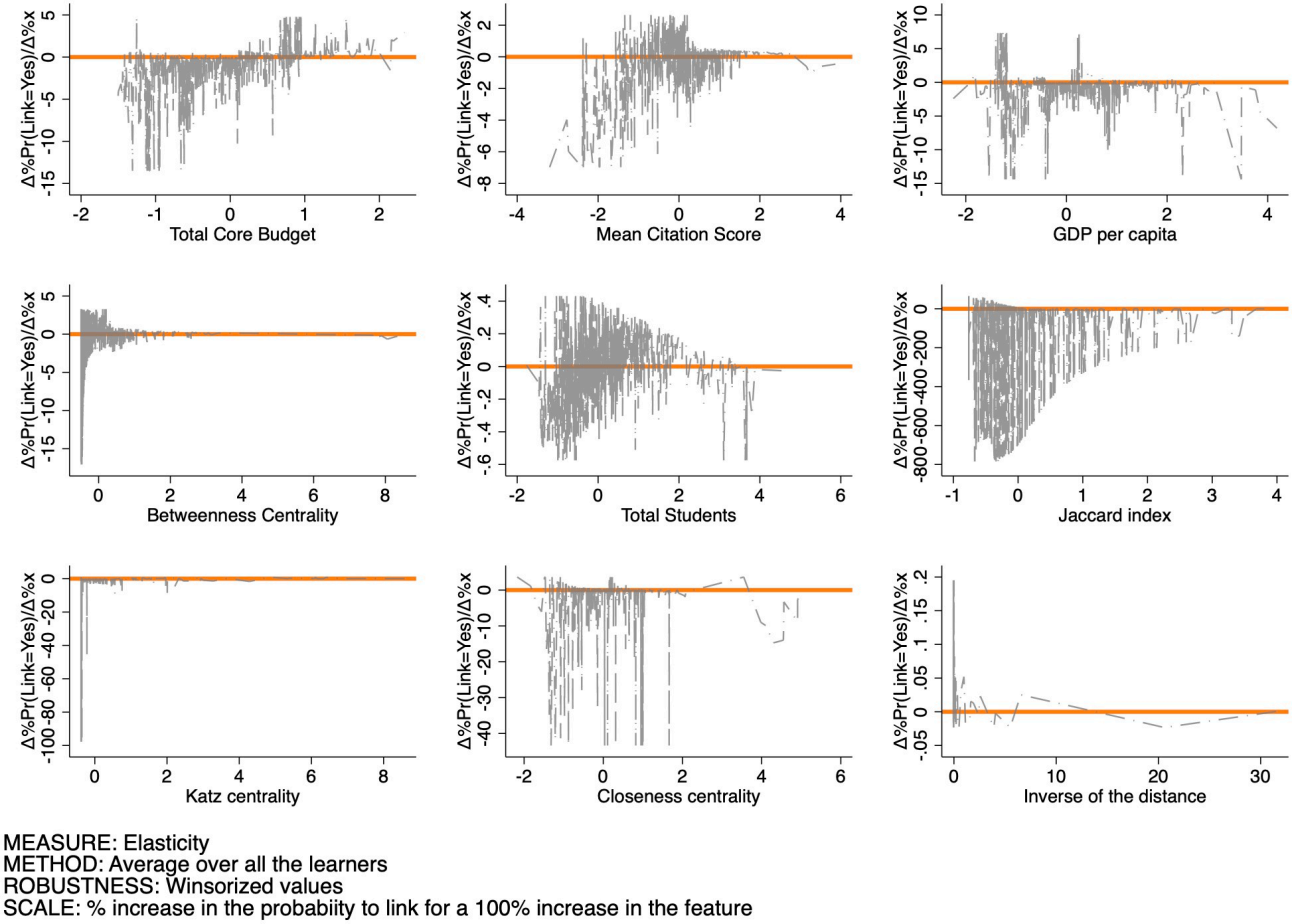
Pattern of the elasticity of the probability of produce a link as a function of the features for LS.

Finally, Figs 15–17 set out the pattern of the elasticity of the probability of forming a link as a function of the features for SSH, PE, and LS respectively. The interpretation of these figures is as follows: for a given feature level $x_{j}^{\prime }$, each point in the graph sets out the percentage increase of the probability to set up a link induced by a 100% increase in the feature evaluated at that specific level. These changes can be both positive or negative, and their variability measures the sensitivity of the link probability to changes in the feature itself. By and large, we observe that: (i) negative elasticities are in general likelier than positive ones over all features and domains; (ii) elasticities are more sensitive to changes in the Betweenness centrality, Closeness centrality, and the Jaccard coefficient than in the other features; (iii) the average sensitivity is larger in the SSH, than in the PE and LS domains.

## Conclusion

Although machine learning methods have been widely used for network link prediction, to the best of our knowledge, our study is the first attempt to predict links using advanced machine learning methods in a competitive project-funding programme, as well as the first to identify the best ML approach in three different ERC domains. Moreover, no other studies have estimated average partial effects within a super-learning setting.

Link prediction is an important challenge in knowledge networks. In this regard, our paper has provided new contributions to this topic. First, we used different analytic procedures to examine and compare the prediction accuracy, both jointly and separately for exogenous and endogenous network variables. Second, we performed data analysis for link prediction in three different ERC domains, i.e. Social Sciences and Humanities, Physical and Engineering Sciences, and Life Sciences.

By jointly using all the selected features, we reached a link prediction accuracy larger than 90% for pretty all the machine learning methods employed. Removing the endogenous features of the nodes (namely, Betweenness centrality, Closeness centrality, the Jaccard coefficient, and Katz centrality) the accuracy drops significantly down in all domains by, on average, 24 percentage points. Furthermore, we observe greater irregular fluctuations for the exogenous variables. Lower accuracy when endogenous factors are removed and higher fluctuation of exogenous ones indicate that predicting connections for the so-called newcomers (for whom we do not know the endogenous features) is significantly more difficult than predicting links for incumbents, who are already in the network (for whom we know both endogenous and exogenous features). Based on our results, Jaccard coefficient appears to have the greatest link predictive power across all scientific domains. As the Jaccard coefficient increases, the probability of forming a link decreases. Sharing common neighboring nodes reduces the likelihood of a connection in joint project collaborations. In SSH and LS, the connection probability falls as the Betweenness grows up. We know that knowledge networks are generally composed of communities connected by links that bridge between them. According to our findings, universities connecting different communities have low link Betweenness. This centrality measure exhibits higher responsiveness in the Social Sciences and Humanities (SSH) domain. The higher responsiveness of Betweenness centrality in SSH implies that its variations have a more pronounced impact on link prediction in this domain.

In the SSH domain, the results appear fairly different than in PE and LS. SSH shows less predictive uncertainty, reflected in the likelihood of forming links. Moreover, elasticities have a wider range of variation in SSH, which accounts for a larger sensitivity to attributes’ change. The size of universities, represented by the total number of students, exhibits higher responsiveness in SSH domain compared to other domains. In SSH, there is a clear pattern where the probability of link formation increases significantly as the size of universities increases. Our results indicate that the Katz and Closeness centralities exhibit lower responsiveness in PE compared to other domains. Compared to other domains, the influence of Katz centrality and Closeness centrality on the likelihood of link creation is less pronounced in PE.

The inverse of distance, which captures the geographic proximity between universities, appears to be important in both PE and SSH domains, but with some differences in their relationship with link prediction. In PE, there is a more linear relationship between the geographic proximity and the probability of link formation. On the other hand, in SSH, the relationship between the inverse of distance and link prediction is not strictly linear; it exhibits an oscillating pattern.

The different ERC domains are characterized by different intellectual and organizational patterns, and cover a wider spectrum of epistemic communities. More heterogeneity of the types of partnerships (in particular with the private sector), and the presence of large laboratories or research infrastructures characterize the PE and LS sectors compared to SSH, and can explain the difference we have found in their link prediction performance.

Our results can be used to explore collaborations on scientific networks between universities by providing useful advice to policy makers. For example, reinforcement mechanisms can be limited in competitive project funding, and policy makers may adopt specific actions with the aim of facilitating integration and boosting the productivity of smaller universities. These actions could include providing targeted support and resources to newcomers to improve their research capabilities and increase their chances of collaboration. This could involve offering funding opportunities specifically designed for newcomers, promoting networking events and conferences to facilitate connections between universities, and fostering partnerships between newcomers (individuals or institutions who are relatively new or less established within the network) and incumbents (experienced researchers or institutions that have been actively involved in the network for a significant period). By implementing such measures, policymakers can create a more inclusive and collaborative research environment, enabling newcomers to contribute effectively to scientific advancements and fostering a more diverse and dynamic scientific community.

Of course, our paper has also limitations. First, the paper analyzes competitive projects funded within the Horizon 2020 programme, whose results cannot be generalized to other science funding programmes. Second, the machine learning methods used in this paper do not explicitly consider the network time serial correlation. We think however that this problem is alleviated in our case by the fact that—within the Horizon 2020 programme—nodes and links are slightly changing over time (differently from what happens, for example, in co-authorship networks). Third, we have considered only collaborations in the Horizon 2020 programme that actually received funding. We did not have information of collaborations generated outside this funding mechanism, thus excluding these network links from our analysis.
